# Properties of Bromine Fused Salts Based on Quaternary Ammonium Molecules and Their Relevance for Use in a Hydrogen Bromine Redox Flow Battery

**DOI:** 10.1002/chem.202103491

**Published:** 2022-02-18

**Authors:** Michael Küttinger, Paulette A. Loichet Torres, Emeline Meyer, Peter Fischer

**Affiliations:** ^1^ Applied Electrochemistry Fraunhofer Institute for Chemical Technology ICT Joseph-von-Fraunhofer Straße 7 76327 Pfinztal Germany; ^2^ Institute for Mechanical Process Engineering and Mechanics Karlsruhe Institute of Technology KIT Straße am Forum 8 76131 Karlsruhe Germany

**Keywords:** bromine, electrolyte, ionic liquids, quaternary ammonium salts, redox flow battery

## Abstract

Bromine complexing agents (BCA) in aqueous electrolytes for hydrogen bromine flow batteries are used to reduce bromine‘s vapour pressure, while an insoluble and liquid fused salt is formed. The properties (concentrations, composition, conductivity and viscosity) of this fused salt are investigated in this study systematically ex situ by using 7 BCAs at different state of charge in HBr/Br_2_/H_2_O electrolytes with a theoretical capacity of 179.6 Ah L^−1^. Bromine is stored in the fused salt at concentrations up to 13.6 M, reaching theoretical volumetrical capacities up to 730 Ah L^−1^ in fused salts. The fused salt consists of a pure, bromine‐ and water‐free ionic liquid of organic [BCA]^+^ cations and polybromides, and its conductivity bases on a hopping mechanism among the polybromides. Alkyl side chain length of the BCAs and distribution of polybromides influence strongly the conductivity and viscosity of the fused salts. 1‐ethylpyridin‐1‐iumbromide results to be favoured BCA for application.

## Graphical abstract

**Properties of fused salts** of quaternary ammonium compounds (QAC) and various polybromides (PB) are investigated for the first time systematically on their applicability in a H_2_/Br_2_ flow battery. Fused salt capacities of up to 730 Ah L^−1^ are reached. Conductivity, which bases on a hopping mechanism between PBs, viscosity and temperature stability depend on the chosen QAC and the fractions of PBs present at a selected state of charge of the electrolyte.

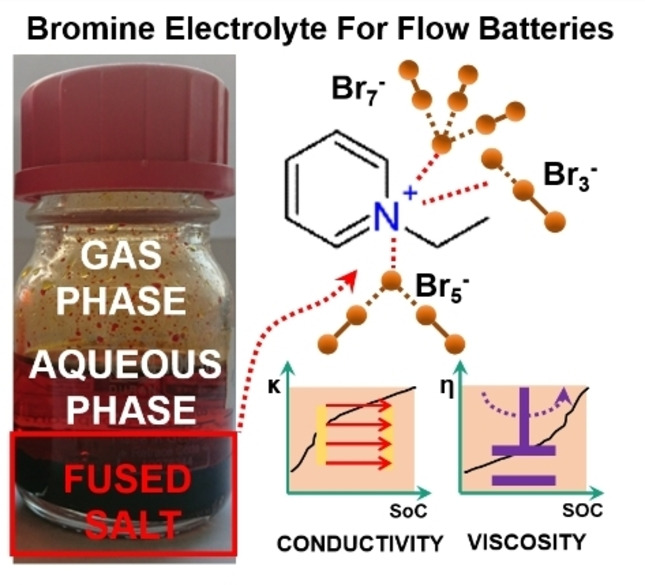

## Introduction

Bromine‐complexing additives (BCA) are salts of organic cations utilised to bind volatile bromine (Br_2_) in aqueous electrolytes of zinc/bromine redox flow batteries (Zn/Br_2_‐RFB) and hydrogen/bromine redox flow batteries (H_2_/Br_2_‐RFB).[^1^, ^2^, ^3^, ^4^, ^5^, ^6^] In these batteries, the positive half cells are operated with bromide‐containing electrolytes, and the reactions are based on the redox couple bromine/bromide.[^7^, ^8^] Electrolytes consist of zinc bromide, supporting electrolytes or HBr and Br_2_ in aqueous solutions and the added BCAs.[^4^, ^5^, ^9^]

The organic cations are usually quaternary ammonium compounds.[^1^, ^4^, ^10^, ^11^] In contact with polybromides in the aqueous solution, they precipitate and form a liquid and heavy fused salt.[^11^, ^12^] The electrolyte consists of an aqueous and a fused salt phase. In this study the properties of these fused salt phases are investigated (conductivity, viscosity, composition) for different 1‐alkylpyridin‐1‐ium bromides and 1‐alkyl‐3‐methylimidazol‐1‐ium bromides with different alkyl side chains (ethyl, *n*‐propyl, *n*‐butyl and *n*‐hexyl), which are shown in Table 1. The influence of the polybromide mixture in the fused salt and of the alkyl side chain length of the BCA on the properties is investigated within the state of charge (SoC) range for an electrolyte with a volumetric capacity of 179.6 Ah L^−1^ for a H_2_/Br_2_‐RFB and impacts on cell operation are discussed.

**Table 1:** Quaternary ammonium halides as BCAs represented by their structure, name and abbreviation for the formation of fused salts and investigation of fused salts properties.

Structure/Substance name and substance abbreviation	Raman shifts of symmetric stretching vibration of polybromides in the fused salt phase	
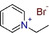	ṽ_Sym._ (Br_3_^−^)	165–159
	ṽ_Sym._ (Br_5_^−^)	253–254
1‐ethylpyridin‐1‐iumbromide, **[C2Py]Br**	ṽ_Sym._ (Br_7_^−^)	267
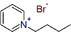	ṽ_Sym._ (Br_3_^−^)	163–161
	ṽ_Sym._ (Br_5_^−^)	254
1‐*n*‐butylpyridin‐1‐iumbromide, **[C4Py]Br**	ṽ_Sym._ (Br_7_^−^)	267
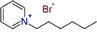	ṽ_Sym._ (Br_3_^−^)	162–160
	ṽ_Sym._ (Br_5_^−^)	252–254
1‐*n*‐hexylpyridin‐1‐iumbromide, **[C6Py]Br**	ṽ_Sym._ (Br_7_^−^)	267
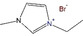	ṽ_Sym._ (Br_3_^−^)	160–159
	ṽ_Sym._ (Br_5_^−^)	254
1‐ethyl‐3‐methylimidazol‐1‐iumbromide, **[C2MIm]Br**	ṽ_Sym._ (Br_7_^−^)	267
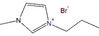	ṽ_Sym._ (Br_3_^−^)	162–160
	ṽ_Sym._ (Br_5_^−^)	254
1‐*n*‐propyl‐3‐methylimidazol‐1‐iumbromide, **[C3MIm]Br**	ṽ_Sym._ (Br_7_^−^)	267
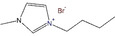	ṽ_Sym._ (Br_3_^−^)	161–160
	ṽ_Sym._ (Br_5_^−^)	253
1‐*n*‐butyl‐3‐methylimidazol‐1‐iumbromide, **[C4MIm]Br**	ṽ_Sym._ (Br_7_^−^)	267
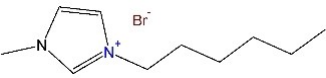	ṽ_Sym._ (Br_3_^−^)	162–160
	ṽ_Sym._ (Br_5_^−^)	254
1‐*n*‐hexyl‐3‐methylimidazol‐1‐iumbromide, **[C6MIm]Br**	ṽ_Sym._ (Br_7_^−^)	267

### State of the art

In the aqueous electrolytes, Br_2_ and bromide form addition compounds[^13^, ^14^] generating polybromide anions Br_2n+1_^−^,[^9^, ^15^] like tribromide (Br_3_^−^), pentabromide (Br_5_^−^) and heptabromide (Br_7_^−^),^[9]^ which are soluble in aqueous solutions.^[1]^ By the addition of [BCA]^+^ cations, these polybromides are extracted into a fused salt phase which operates as a bromine and energy storage reservoir.^[6]^ While bromide salts of many [BCA]Br are highly soluble in aqueous solutions,^[2]^ there is poor solubility of the BCA‐polybromide form in the aqueous phase.[^2^, ^16^] The solubility equilibrium is shown in Equation (1). The fused salt precipitates to form an additional, heavy and liquid electrolyte phase.[^1^, ^2^, ^4^, ^10^, ^17^]

(1) $${\left[BCA\right]}^{+}\left(aq\right)+\;{{Br}_{2n+1}}^{-}\left(aq\right)\vec{\leftarrow }\;\left[BCA\right]{Br}_{2n+1}↓\left(fs\right)$$

Mainly investigated BCAs are from the groups of 1‐alkylpyridinium halides, 1‐alkyl‐3‐methylimidazolium halides, 1‐alkyl‐1‐methylpyrrolidinium halides, 1‐alkyl‐1‐methylmorpholinium halides or aliphatic ammonium compounds with ethyl, *n*‐butyl or *n*‐hexyl side chain groups.[^1^, ^2^, ^4^, ^5^, ^10^, ^11^, ^18^, ^19^, ^20^, ^21^, ^22^, ^23^, ^24^] In the past BCAs have been mainly applied and investigated in Zn/Br_2_‐RFB electrolytes.[^4^, ^12^, ^18^, ^20^, ^21^, ^23^, ^25^, ^26^]

The vapour pressure and volatility of Br_2_ in the aqueous phase are significantly reduced by the use of the BCAs.[^16^, ^18^, ^21^, ^27^] They potentially increase the safety towards the toxicity of bromine gas[^18^, ^28^] for operators when handling the bromine‐containing electrolytes and increase the solubility of Br_2_ in the electrolyte.^[2]^ It is known that BCAs bind bromine of the electrolyte strongly in the fused salt phase, while only low Br_2_ concentrations remain in the aqueous phase, depending on the composition of the electrolyte and the type of BCA.[^2^, ^4^] During the operation of the H_2_/Br_2_‐RFB with a BCA, aqueous electrolyte phases have been pumped through the cell.[^5^, ^29^] Reported concentrations of Br_2_ in the aqueous phase range between 33 and 163 mM as initially used in Zn/Br_2_ electrolytes.^[10]^ However, this concentration range can be considered too low and limit the discharge performance of the battery.[^12^, ^20^] Low Br_2_ concentrations between 5.5 and 313 mM are also found in the aqueous phase of BCA‐containing electrolytes for H_2_/Br_2_‐RFB.^[2]^ Therefore, increasing interest in the possibility of using the remaining high concentrated Br_2_ fused salt phases in the positive half‐cell of a bromine RFB is ambitioned.^[12]^

Application of the fused salt phase in Zn/Br_2_‐RFB systems is described in literature.[^4^, ^11^, ^16^, ^24^, ^30^] The use of BCA results in a low self‐discharge rate in the RFB as crossover of Br_2_ is limited,^[6]^ which improves the coulomb efficiency of the battery.[^18^, ^20^, ^21^, ^31^] In general, limiting the crossover of Br_2_ with the BCA‐containing electrolytes, also prevents the possibility of corroding the active materials of the corresponding negative half cells of the battery. Hence, it prevents parasitic reactions such as Zn corrosion on Zn/Br_2_‐RFBs[^4^, ^20^, ^31^] and Pt corrosion on the hydrogen half cell of the H_2_/Br_2_‐RFBs.[^8^, ^32^] An additional advantage is that fused salt drops adhering to the electrode surface lead to higher discharge current densities, since Br_2_ is released from the fused salt into the aqueous phase directly at the electrode surface.^[12]^ Amit et al.^[33]^ apply emulsions of aqueous electrolyte and the fused salt in a membrane‐less Zn/Br_2_‐RFB. In their work they show that increasing the volume fraction of the fused salt phase from 1 to 5 vol % in the emulsion, significantly increased both the discharge current densities and the ohmic resistance of the cell.^[33]^ If not managed correctly, the accumulation of fused salt droplets on the graphite electrode surfaces^[34]^ can lead to the complete coverage of the electrode surface which results in a decrease of the current densities of the cell.^[12]^ Additionally, Magnes et al.^[4]^ use fused salt in a membrane‐free Zn/Br_2_‐RFB, while the set‐up consisted of a horizontally positioned cell that allowed the heavy fused salt to be overlaid with aqueous electrolyte. With a strong BCA‐cation, such as 1‐*n*‐butyl‐3‐methylimidazol‐1‐ium, the deposition efficiency of Zn is thereby increased in the zinc half cell.^[4]^

Viscosities of the fused salt in ZnBr_2_ electrolytes between 50 and 70 mPas and conductivities between 50 and 60 mS cm^−1^ are reported in literature,^[34]^ while other authors found conductivities between 63 and 115 mS cm^−1^ (calculated from Ref. [23]). Different BCAs in various electrolyte mixture have been investigated.^[23]^

Although previous literature shows that fused salt phases are used especially in Zn/Br_2_‐RFB to improve discharge performance, only rare information about their composition, Br_2_ concentration in combination with their influence on fused salt conductivity and viscosity have been published. For high concentrated electrolytes for H_2_/Br_2_‐RFB no data for fused salt properties are accessible in literature. All parameters are indispensable for designing a RFB system including fused salts. Further the composition and anion characters of the BCA‐polybromide fused salts has also not been conclusively clarified to date,[^12^, ^22^] although different polybromide anions up to Br_11_^−[10,12]^ or mixtures of BCA and Br_2_ ^[22]^ are assumed. Influence of BCAs, their structure and alkyl side chain length as well as composition of the fused salt for high capacity electrolytes in H_2_/Br_2_‐RFB has not been investigated, leading to a lack of background information for BCA application in this battery type.

### Working plan

In this work, the heavy fused salt phases of 7 BCA‐containing HBr/Br_2_/H_2_O electrolytes are systematically investigated on their properties for the first time. The polybromide fused salts of the BCAs 1‐ethylpyridin‐1‐ium bromide [C2Py]Br, 1‐*n*‐butylpyridin‐1‐ium bromide [C4Py]Br, 1‐*n*‐hexylpyridin‐1‐ium bromide [C6Py]Br, 1‐ethyl‐3‐methylimidazol‐1‐ium bromide [C2MIm]Br, 1‐*n*‐propyl‐3‐methylimidazol‐1‐ium bromide [C3MIm]Br, 1‐*n*‐butyl‐3‐methylimidazol‐1‐ium bromide [C4MIm]Br and 1‐*n*‐hexyl‐3‐methylimidazol‐1‐ium bromide [C6MIm]Br are tested ex situ on their properties within the entire SoC range in an electrolyte with a storage capacity of 179.6 Ah L^−1^. The structures of this BCAs are shown in Table 1.

**Figure 1:**
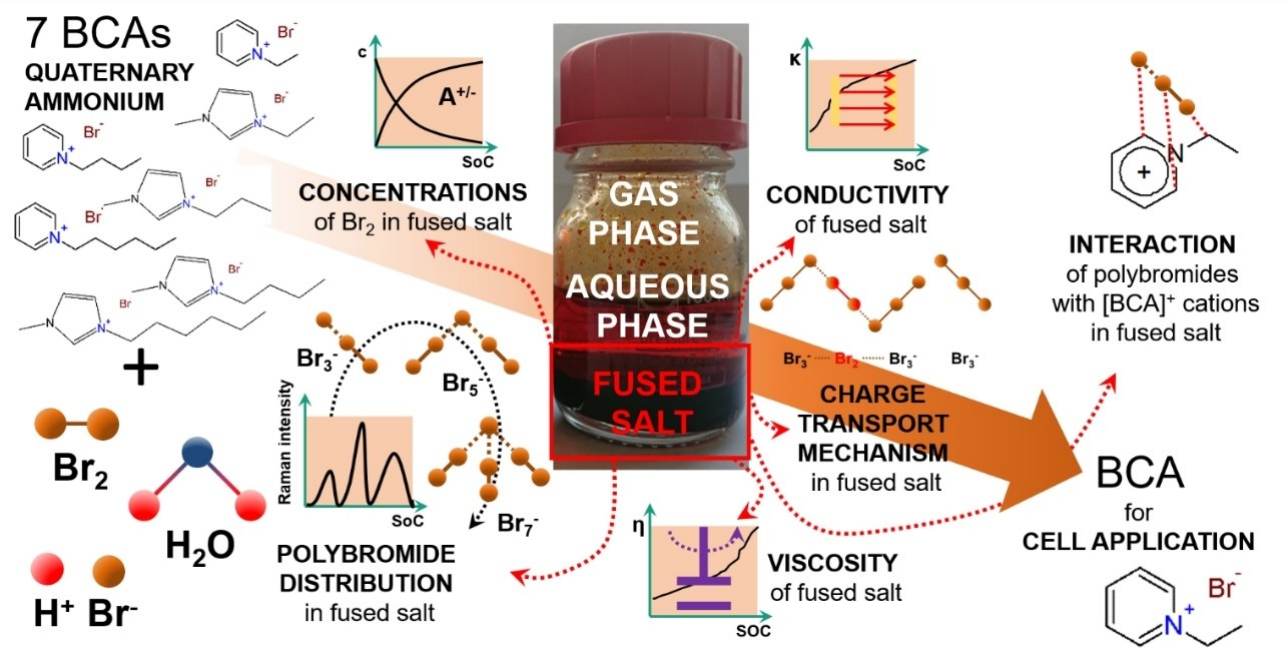
Scheme of the procedure to investigate properties of the fused salt phase(fs) from 7 BCAs with different alkyl side chain groups at pyridine and 3‐methylimiazole basic molecule structure: Concentration of Br_2_ (fs), distribution of Br_2_ in different polybromides Br_3_^−^, Br_5_^−^ and Br_7_^−^, conductivity (fs) and charge transport mechanism depending on fused salt composition, viscosity (fs) and explanation of binding strength between the BCAs and the polybromides in the fused salt. One BCA out of 7 BCAs is selected on the basis of the results for feasible application in H_2_/Br_2_‐RFB.Results and Discussion. The photo in the scheme shows an example of a bromine electrolyte with the well‐known BCA 1‐ethyl‐1‐methylpyrrolidin‐1‐ium bromide [MEP]Br,[^10^, ^12^, ^20^, ^21^, ^23^, ^26^] with the heavy fused salt on the bottom of the glass, overlaid by the aqueous electrolyte phase and on top the gas phase. (Drops in the gas phase are aqueous phase drops at the glass from shaking the two phase electrolyte.)

This study complements the work of Küttinger et al.^[2]^ which discusses in detail the properties of the aqueous electrolyte phase for use in H_2_/Br_2_‐RFB. The corresponding properties of the fused salt phase for the same BCAs are compared and discussed here. A scheme of the work plan is shown in Figure 1. The selection of the investigated BCAs is based on results presented in Ref. [2].

For the first time, bromine concentrations in the fused salt phase and volumetric storage capacities of the fused salt phase are determined. At the same time, the exact composition of the fused salt phases based on ingredients and their fractions are determined by help of Raman spectroscopy. Based on the knowledge of the exact compositions of the fused salt, conductivities and viscosities of the fused salt phase are determined and discussed. The focus is on the influence of the [BCA]^+^ cation structure and composition at various state of charge (SoC) on the parameters. An adapted mechanism for the conductivity of the fused salt phase is proposed based on originally proposed mechanisms and a possible application scenario of fused salt in H_2_/Br_2_‐RFB cells is presented.

## Results and Discussion

### Concentration of Br_2_ in the fused salt phase

#### Fused salt fraction in the electrolyte, Br_2_ fused salt concentration and energy capacity of the fused salt phase

**Figure 2:**
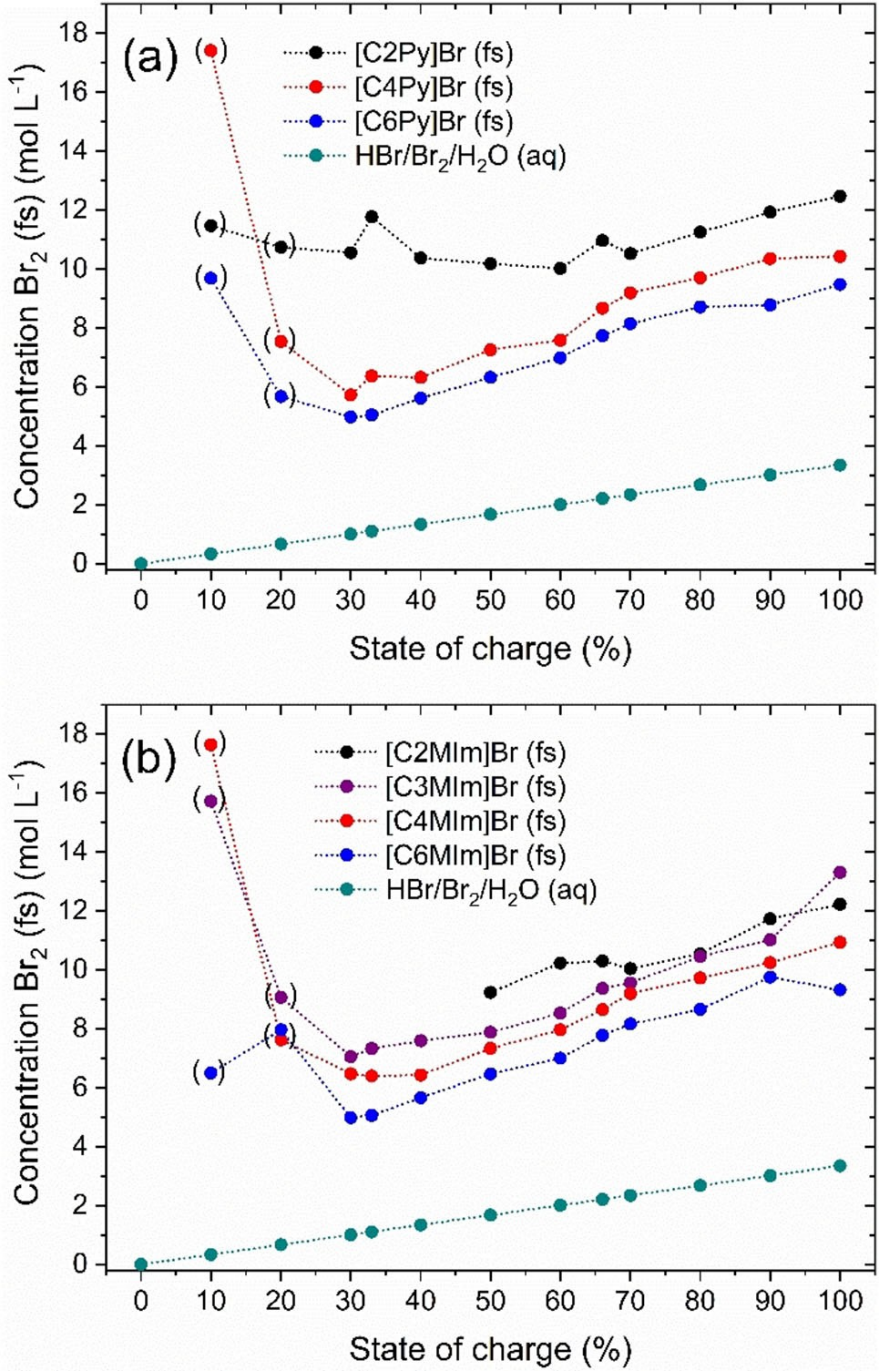
Concentrations of Br_2_ in the liquid fused salt as a function of the state of charge (SoC) and the investigated BCAs with (a) 1‐alkylpyridin‐1‐ium bromides and (b) 1‐alkyl‐3‐methylimidazol‐1‐ium bromides as BCAs with different alkyl side chains in the N‐position and comparison with the total Br_2_ concentration (green) in the two‐phase electrolyte sample.

High Br_2_ concentration of fused salts are of interest for their application in the positive half‐cell during discharge, where Br_2_ is reduced to bromide (Br^−^). As discussed by Fabjan et al.^[12]^ the use of fused Br_2_ salts increased discharge current densities and power densities of the RFBs. Mass transport limitations or limitations due to insufficient convection of Br_2_ in front of the electrode are expected to be reduced compared to aqueous solutions with low concentrated Br_2_. From Ref. [2] the Br_2_ concentrations in the aqueous phase are known to be quite low with a maximum of 11 mol% Br_2_ stored there. As a comparison, the determined concentrations of Br_2_ in the fused salt are shown in Figure 2.

From our previous publication, we know that at least 89 mol% of the Br_2_ is transferred to the fused salt phase^[2]^ where it is stored compactly in rather small volumes. Br_2_ is mainly stored in a reddish‐brown, liquid and heavy second phase, as shown in the centred photo in Figure 1. For SoC<30 %, the measured volumes of the fused salts are smaller than 2 mL, leading to errors in the determination of the volumes and Br_2_ concentrations. This range is not considered in the detailed discussion. Volume fractions of the fused salt in the electrolyte are in the range between 7.5 and 27.7 vol % as shown in Table S5 in the Supporting Information.

The fraction of fused salt increases with increasing SoC for all applied BCAs. Due to the rising absolute concentration of Br_2_ in the electrolytes, Br_2_ is increasingly stored in the fused salt and its volume fraction increases. Using the example of the fused salt based on [C2Py]Br_2n+1_(fs), its volume fraction increases from 7.5 vol % at SoC=30 % to 22.1 vol % at SoC=100 %. This tendency is observed for all investigated BCAs and is tabulated in the Supporting Information (Table S5). Simultaneously, the size of the [BCA]^+^ cation influences the volume fraction of the fused salt in the electrolyte. The larger the [BCA]^+^ cation or the longer the *n*‐alkyl side chain, the larger the volume fraction of the fused salt in the total electrolyte volume at same SoC values. Using the example of the electrolyte at SoC=50 %, the volume fraction of the fused salt increases from 13.2 vol % for [C2Py]Br_2n+1_(fs) via 18.3 vol % for [C4Py]Br_2n+1_(fs) to 20.8 vol % for [C6Py]Br_2n+1_(fs). Volume fractions between 1‐alkylpyridin‐1‐ium‐BCAs and 1‐alkyl‐3‐methylimidazol‐1‐ium‐BCAs both having the same *n*‐alkyl side chain and at the same SoC differ only slightly and there is no distinct trend for the influence of the two different structures of the BCAs.

The Br_2_ concentrations in the fused salt c(Br_2_, fs) with 4.98≤c(Br_2_, fs)≤13.62 M are significantly higher than the total, theoretical concentration of Br_2_ in the electrolyte mixture with a maximum of 3.35 M Br_2_ at SoC=100 % (Figure 2a and b green line/dots). Br_2_ concentrations in the fused salts are tabulated in Table S3 in the Supporting Information. In comparison to the Br_2_ concentrations in the aqueous phase with c(Br_2_, aq)<0.35 M from Ref. [2] for the same samples, the huge difference in concentration between the two phases is noticed, showing the strong bromine binding strength of the BCA cation in contact with polybromides. The solubility equilibrium (Eq. (1)) is strongly shifted in favour of the salt formation of [BCA]Br_2n+1_(fs). Based on the obtained results, and considering the amount of Br_2_ stored in the fused salt only, capacities ranging between 266.9 Ah L^−1^ and 730.0 Ah L^−1^ can be achieved.

In this sense, the fused salt phase represents a high‐density energy storage solution for the positive half‐cell of the H_2_/Br_2_‐RFB. Although the volume fraction of the fused salt increases with rising SoC due to the uptake of Br_2_ and [BCA]Br, tabulated in Table S5 in the Supporting Information, the concentration of Br_2_ in the fused salt increases between 30≤SoC≤100 %. Bromine accumulates progressively in this phase. As a result, increasingly more energy from the positive half‐cell is stored in the fused salt in a smaller phase volume.

However, it should be explicitly pointed out here that an operation of the positive half‐cell in the H_2_/Br_2_‐RFB with pure fused salt is not possible. The aqueous electrolyte solution contains and transports protons into the positive half‐cell or takes up protons from there, depending on the operation mode. The aqueous electrolyte solution is therefore essential for the half cell reaction of the negative hydrogen half‐cell.

**Figure 3:**
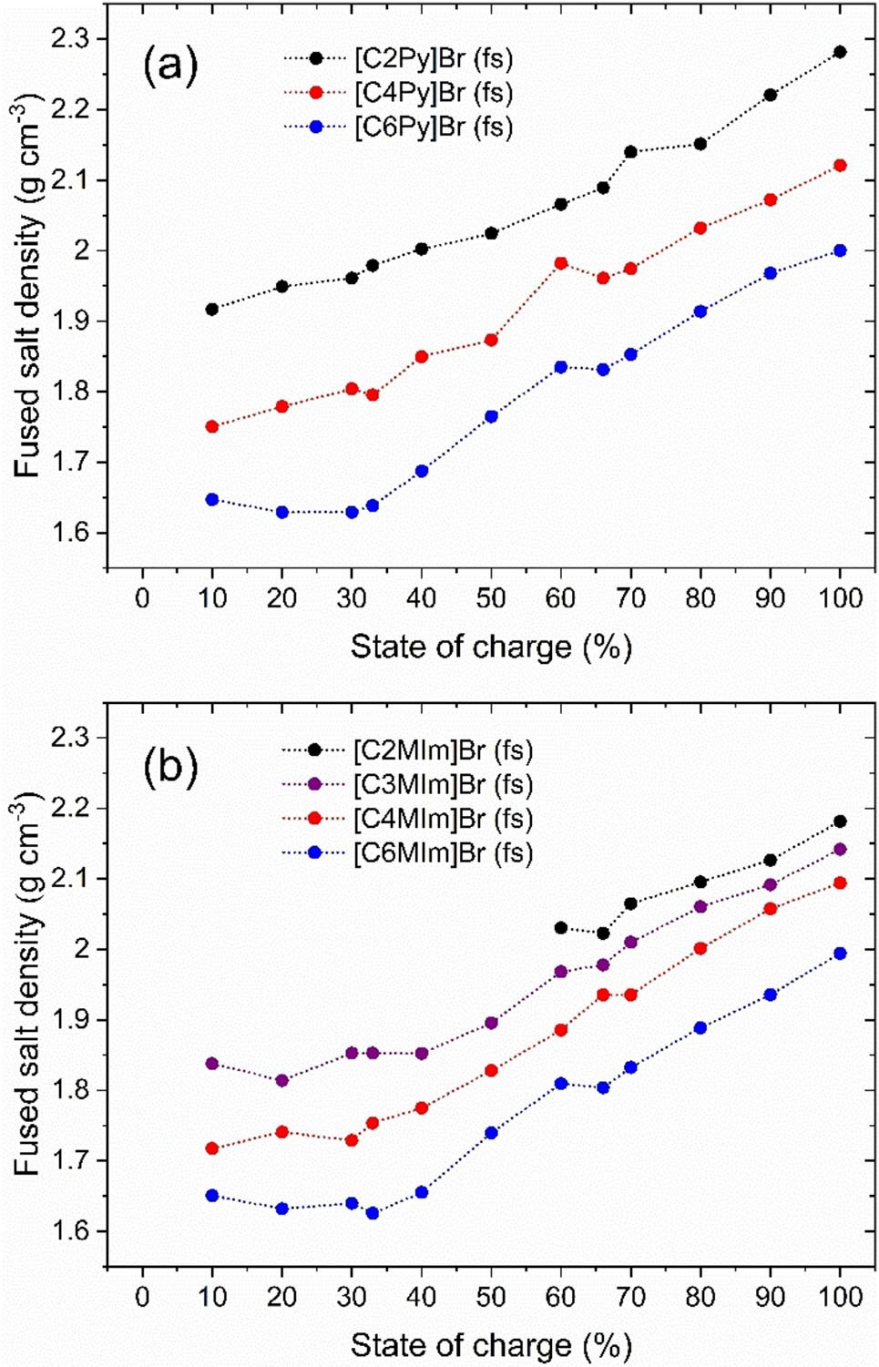
Densities of the fused salt phases depending on the SoC of the electrolyte and of the chosen [BCA]^+^ cation with different alkyl side chain length with (a) 1‐alkylpyridin‐1‐ium cations and (b) 1‐alkyl‐3‐methylimidazol‐1‐ium in the fused salt at ϑ=23±1 °C.

The fact that increasingly more Br_2_ is stored in the fused salt in a smaller volume with increasing SoC is confirmed by rising densities of the fused salt at ϑ=23±1 °C shown in Figure 3.

This parameter is an independently determined value which confirms the increase in concentration of Br_2_ in the fused salt phase. The measured fused salt densities ρ(fs) are tabulated in Table S6 in the Supporting Information and show high values ranging between 1.63≤ρ(fs)≤2.28 g cm^−3^. In comparison, pure bromine holds a density of 3.12 g cm^−3^ at room temperature.^[35]^ The density of the fused salt phase strongly depends on the SoC, i. e. the composition of the fused salt phase, and at the same time strongly on the [BCA]^+^ cation present in the fused salt. In principle, this trend is consistent with the trend of the bromine concentration in the fused salt shown in Figure 2.

On the basis of the high bromine concentrations, the fused salt would be ideally suited for use in the cell during the discharge process, since high bromine concentrations are locally available for the bromine reduction reaction. Based on the results in Figure 2, [C2Py]Br is the most promising candidate for application in the cell. Compared to the other BCAs investigated, the associated fused salt of the [C2Py]Br remains stable as a liquid throughout the entire SoC range and simultaneously achieves one of the highest and most stable concentration in the fused salt evaluated. As seen in Figure 2a the concentration range of the [C2Py]Br (fs) changes with the SoC in a range between 11.47≤c(Br_2_,fs)≤12.47 M which translates into to a capacity of 615 Ah L^−1^ to 668 Ah L^−1^ just in the pure fused salt phase of the electrolyte. Additionally, the density of the fused salt phase of [C2Py]^+^ is highest in direct comparison to that of other [BCA]^+^ cations in the investigated SoC range with 1.92 ≤ ρ(fs) ≤ 2.28 g cm^−3^. Moreover, it was shown that the aqueous phase of [C2Py]Br also presents high conductivities,^[2]^ moderate Br_2_‐sequestration^[31]^ and low polarization of the cell,^[20]^ which makes the use of this BCA even more suitable for RFB applications.

#### Influence of the BCA basic molecule structure and side chain length on fused salt Br_2_ concentration and phase density

Overall, the concentration of Br_2_ in the fused salt depends on the chosen BCA. Short alkyl side chain length in the BCA leads to rising Br_2_ concentrations in the fused salt independent of the SoC (see Figure 2). It is known from Küttinger et al.^[2]^ that same BCAs with long side chains have a much higher binding strength towards Br_2_ compared to short alkyl side chain BCAs. However, based on the trend observed in Figure 2, the concentration of Br_2_ for the long and short side chain alkylated BCAs is dominated by the effect of the BCA cation size in the fused salt. More specifically, smaller organic BCA cations lead to higher concentrations of Br_2_ in the fused salt phase. This is expected to be because the organic BCA molecules increase in size with increasing chain length of their alkyl side group, thus, requiring larger volumes in the fused salt. Therefore, an increase in volume due to increasing BCA molecule size leads to lower molar concentrations of Br_2_ in the fused salt phase. The size of the [BCA]^+^ cation in parallel influences the density of the fused salt phase (see Figure 3) caused by the same effects. The overall trend shows that the long side chain BCA cations lead to lower fused salt densities compared to short side chain BCA cations.

Additionally, we observed that the Br_2_ concentrations appear to be independent of the selected basic molecule structure of the BCAs (pyridine or 3‐methylimidazole). Fused salts out of both groups with equal lengths of the alkyl side chains lead to approximately equal bromine concentrations (see Figure 2 and values in Table S3 of the Supporting Information). With this, we can conclude that the length of the alkyl side chain is the decisive factor affecting the concentration of Br_2_ in the fused salt phase. On the other hand, the densities of the investigated BCAs depend on both the basic structure and the side chain length of the BCA. In general, the density of the fused salts for the 1‐alkylpyridin‐1‐ium‐BCAs are 0.01 to 0.1 g cm^−3^ larger than the densities obtained for 1‐alkyl‐3‐methylimidazol‐1‐ium BCAs. The lowest observed differences are for [C6Py]Br and [C6MIm]Br, while for [C2Py]Br and [C2MIm]Br the densities differ the most out of the BCAs investigated. For [C2Py]Br and [C2MIm]Br the difference of fused salt density is caused by having one ethyl side chain group in [C2Py]Br, while one ethyl and one methyl side chain are available in [C2MIm]Br, leading to a larger molecule size and to a reduced density compared to fused salts of [C2Py]Br.

Within the SoC range from 30 % to 100 %, the concentrations of Br_2_ in the fused salt increase. At the same time, the total bromine concentration in the electrolytes also increases linearly from 0 M at SoC=0 % to 3.35 M at SoC=100 % due to the oxidation reaction of bromide to bromine in the cell. Since the volume of the fused salt phase also increases, an increase in concentration in the fused has to imply that in this case the storage form of Br_2_ is increasingly compact. The measurements of the fused salt density, shown in Figure 3, confirm the concentration measurements and the more compact storage of [BCA]Br_2n+1_(fs). This is determined in detail by examining the distribution of Br_2_ among the different polybromides in the fused salt described in the following section.

### Composition of the fused salt phase and bromine binding strength of BCA in the fused salt

The change of the bromine concentration and the density of the fused salt phase as a function of the SoC indicates changes in the composition of the fused salt phase with the SoC, probably affected by the formation of polybromides. However, hardly any information is available in the literature about the composition of these fused salt phases. In order to gain a deeper insight into this topic and to elucidate the different compositions of the investigated phases, Raman spectra of the fused salt samples are recorded between a Raman shift of 100≤$\tilde{\nu }$ ≤4000 cm^−1^. Overall, the knowledge about the fused salt composition allows an understanding of Br_2_ storage properties considering the solubility equilibrium Equation (1), the fused salt conductivity and viscosity investigated in the subsequent sections.

#### Existing components in the fused salt

Comprehensive information about the composition of the fused salt phase can be determined from the Raman spectra. For all investigated BCAs, the fused salt is a pure and water free ionic liquid based on [BCA]^+^ cations and polybromide anions Br_2n+1_^−^, free of pure Br_2_ molecules within the whole SoC range of the electrolyte. The polybromide anions present in the fused salt have been reported in a previous work^[2]^ to be tribromide Br_3_^−^, pentabromide Br_5_^−^ and heptabromide Br_7_^−^ for fused salts of [C3MIm]^+^, [C4MIm]^+^ and [C6MIm]^+^ cations at SoC=33 %.^[2]^

**Figure 4:**
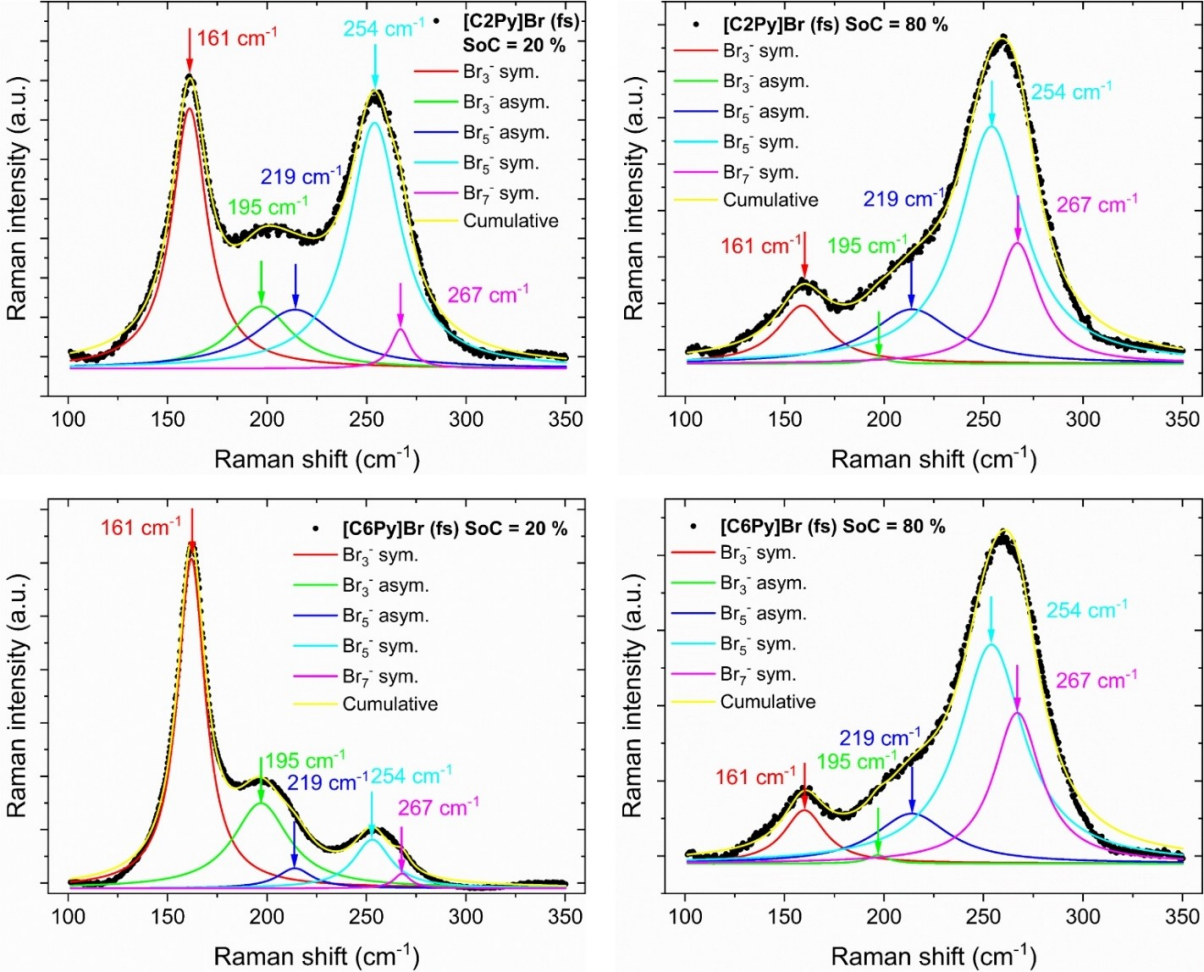
Raman spectra of different fused salt phases (black dots), and fitting results for the symmetric and antisymmetric stretching vibrations of Br_3_^−^, Br_5_^−^ and Br_7_^−^, as well as the sum of all Raman fits: (a) fused salt of [C2Py]^+^ cations at SoC=20 %, (b) [C2Py]^+^ cations at SoC=80 %, (c) [C6Py]^+^ cations at SoC=20 % and (d) [C6Py]^+^ cations at SoC=80 %.

An example of the recorded Raman spectra can be found in Figure 4 and for all BCAs and SoCs in the Supporting Information in Tables S1 to S7. Initially, the Raman spectra have been investigated on characteristic peaks caused by water molecules, as previous work by Skyllas‐Kazacos^[22]^ described the fused salt to be an anhydrous oily phase. The presence of water can be easily identified by Raman spectroscopy as the expected Raman shifts of water in aqueous HBr solutions is set to occur at $\tilde{\nu }$ ≈1600 cm^−1^ and between $\tilde{\nu }$ ≈3000 cm^−1^ and 3800 cm^−1^.^[36]^ As expected, the Raman spectra of the fused salts show no characteristic Raman shifts of water for all BCAs evaluated within the whole SoC range (results not shown). In addition, Raman experiments gave no evidence on the presence of free Br_2_ in the fused salts. The Raman activity of pure Br_2_ molecules is generally evidenced by a sharp single peak or shoulder at a Raman shift between 300 cm^−1^≤$\tilde{\nu }$ ≤320 cm^−1^[^14^, ^37^, ^38^, ^39^], however this signal is not detected for any of the chosen BCAs of the evaluated SoCs.

While the investigated aqueous electrolytes had a total concentration of 1.11 M [BCA]Br, the concentration of the BCAs in aqueous solutions can reach much higher values. As an example, [C2Py]Br solutions with concentrations in pure water of 6.57 M at ϑ=23±1 °C can be reached, while in 48 wt % (8.7 M) HBr solution still 5.68 M [C2Py]Br are soluble. The solubility of bromide is predominant in the form of [BCA]Br(aq) and also HBr tends to be solvated in aqueous solution due to its pKs≈−8.2 to−9,^[40]^ while polybromides in contact with [BCA]^+^ are hardly soluble in the aqueous phase. We suggest that due to the outstanding solubility of [BCA]Br and HBr in aqueous solutions, both substances stay solvated in the aqueous phase and are not transferred into the fused salt phase.

The Raman peaks of the polybromides Br_3_^−^, Br_5_^−^ and Br_7_^−^, are investigated in the wavenumber range between 100≤$\tilde{\nu }$ ≤350 cm^−1^. Additionally, Figure 4 includes the fitting results for the different polybromide stretching vibrations by the example of fused salts based on [C2Py]Br and [C6Py]Br at SoC=20 and 80 %. All Raman spectra are provided in the Supporting Information in Figure S1 to S7 for the investigated fused salts and SoCs. Characteristic Raman wavenumbers of the different polybromide Raman vibrations in aqueous solution from literature are applied to determine fused salt polybromides and show the presence of Br_3_^−^, Br_5_^−^ and Br_7_^−^ in the fused salt phase: $\tilde{\nu }$ (Br_3_^−^, sym.)≈164–170 cm^−1^,[^13^, ^41^, ^42^] $\tilde{\nu }$ (Br_3_^−^, antisym.)≈190–198 cm^−1^,[^13^, ^37^, ^42^] $\tilde{\nu }$ (Br_5_^−^, antisym.)≈210 cm^−1^,[^13^, ^42^] $\tilde{\nu }$ (Br_5_^−^, sym.)≈253–255 cm^−1[13,37–39,42]^, $\tilde{\nu }$ (Br_7_^−^, sym.)≈269 cm^−1^.[^13^, ^38^]

Raman shifts of the polybromide stretching vibrations are slightly lower (max. 5 cm^−1^) in the fused salts compared to their equivalents in the aqueous phase (from Ref. [2]), and are tabulated in Table 1. The difference between the observations in the aqueous and fused salt phase might depend on the lack of solvent/water solvation in the fused salt phase. Thus, a solvate shell does not enclose the molecules in the fused salt. Raman shifts of polybromide vibration in the aqueous phase of the same BCAs are tabulated in Ref. [2]

Figure 4 presents as an example a comparison of two BCAs with different lengths of the alkyl side chains ([C2Py]Br and [C6Py]Br) under two different SoCs (20 and 80 %). While for SoC=20 % the peaks of Br_3_^−^ stretching oscillation in the fused salt of [C6Py]Br strongly dominate compared to the peaks of the higher polybromides Br_5_^−^ and Br_7_^−^, for the fused salt phase of [C2Py]Br the peaks of Br_3_^−^ and Br_5_^−^ are present in similar proportions. At low SoCs the fused salt of [C6Py]Br consists mainly of tribromide salt [C6Py]Br_3_ while in [C2Py]Br based fused salt mixtures of tri‐ and pentrabromide salts [C2Py]Br_3_ and [C2Py]Br_5_ are available. (The detailed fractions of Br_2_ in polybromides are discussed in the following subchapter.) It shows that bromine is stored and bound in [C2Py]Br fused salts in both polybromides, while for the longer *n*‐hexyl side chains in [C6Py]Brfused salts, storage takes place primarily in the form of Br_3_^−^. As known from Ref. [2], [C6Py]Br is a much stronger Br_2_‐binding BCA than [C2Py]Br. We can validate this by the fact that [C6Py]^+^ cations combined with tribromide are hardly soluble in the aqueous phase and pass into the fused salt as shown here. [C2Py]^+^ cations are more soluble, but also accumulate with pentabromide during precipitation. The combination of BCAs and tribromide leads to energetically more favourable states and depends strongly on the chosen BCA.^[42]^

For SoC=80 %, on the other hand, the peak dimensions for Br_3_^−^ and Br_5_^−^ hardly differ at first between the two BCAs, but a storage of Br_2_ in the form of Br_7_^−^ seems slightly to be more pronounced in the fused salt of [C6Py]Br than in the fused salt of [C2Py]Br. However, the differences between the spectra at SoC=80 % are basically rather small, so that it can be assumed that at SoC=80 % only a small dependence of the Raman spectra and the distribution of Br_2_ on the polybromides (next subchapter) on the BCA is to be expected.

Between SoC=20 % and SoC=80 %, Br_2_ is increasingly stored in Br_5_^−^ and Br_7_^−^, while the peaks of Br_3_^−^ are proportionally very small. For high SoCs, Br_2_ is predominantly stored in the form of Br_5_^−^ and Br_7_^−^.

In summary, the fused salt phase is composed purely of [BCA]^+^ cations and various polybromides, with the presence of the individual polybromides depending on the SoC. An anhydrous ionic liquid is present which is also free of pure bromine. One organic [BCA]^+^ cation per polybromide anion is present in the fused salt phase to keep the charge balance in the fused salt settled.

#### Distribution of Br_2_ in the different polybromides in the fused salts

**Figure 5:**
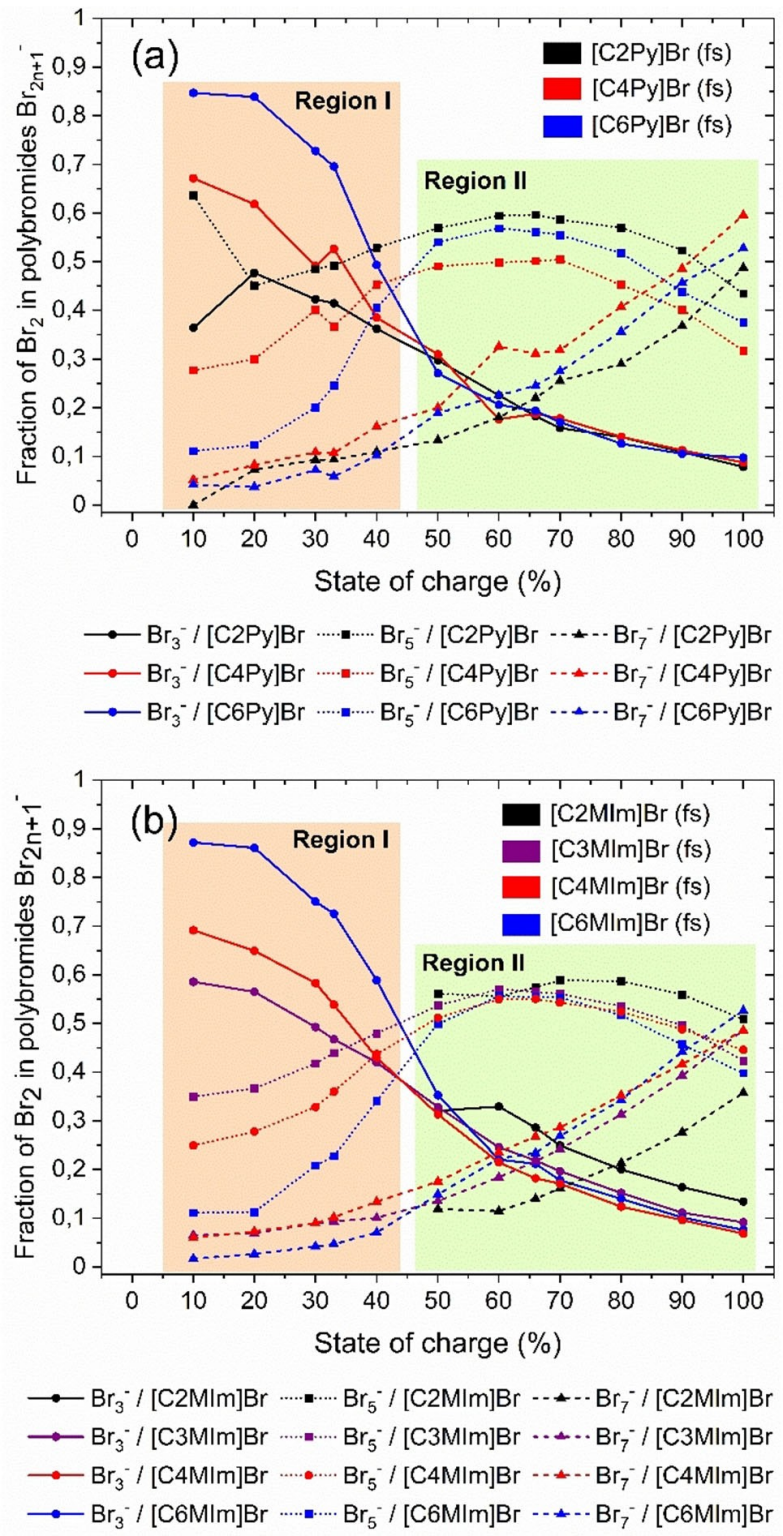
Distribution of Br_2_ in the fused salt to polybromides Br_3_^−^, Br_5_^−^ and Br_7_^−^ with different alkyl side chains in the N‐position of the BCA with (a) 1‐alkylpyridine‐1‐ium bromides and (b) 1‐alkyl‐3‐methylimidazole‐1‐ium bromides as BCAs at ϑ=23±1 °C.

In order to obtain the distribution of Br_2_ among the different polybromides in the fused salt phases depending on the SoC and the [BCA]^+^ cation, the fractions of Br_2_ in the polybromides x(Br_3_^−^), x(Br_5_^−^) and x(Br_7_^−^) found for the respective SoC are calculated based on the Raman spectra. Investigation of the distribution is described in the “Experimental section” and is discussed in Ref. [9] and Ref. [2] The results are summarized in Figure 5 for the investigated BCAs, 1‐alkylpyridin‐1‐ium bromide and 1‐alkyl‐3‐methylimidazol‐1‐ium bromide.

For SoC<50 % (Figure 5, region I), a fraction of 80 to 90 mol% of the Br_2_ is stored in Br_3_^−^ and Br_5_^−^ for all fused salts. In addition, there is a strong dependence between the distribution of Br_2_ in Br_3_^−^ and Br_5_^−^. For SoC≤33 %, all BCAs in this range in the fused salt preferably form [BCA]Br_3_(fs) rather than [BCA]Br_5_(fs). Br_2_ bound in Br_7_^−^ is hardly present at small SoCs with x(Br_7_^−^)<11 mol%. The storage of Br_2_ in certain polybromides in the fused salt also depends on the length of the alkyl side chain in N‐position of the BCA molecules. BCAs with larger alkyl side chain length form fused salts based on Br_3_^−^ anions while BCAs with shorter alkyl side chain form fused salts based on a mixture of Br_3_^−^ and Br_5_^−^ in this SoC range. Br_2_ and Br^−^ in contact with [C6MIm]^+^ and [C6Py]^+^ form [C6MIm]Br_3_ and [C6Py]Br_3_ with a fraction between 69.5≤x(Br_3_^−^)≤87.2 mol% for SoC≤33 %. For [C4MIm]^+^, [C4Py]^+^ and [C2Py]^+^ and [C3MIm]^+^ still main fractions of Br_2_ with 41.4≤x(Br_3_^−^)≤69.2 mol% are stored in [BCA]Br_3_ in fused salts, but the fraction of Br_2_ stored in [BCA]Br_5_ increases with 27.7≤x(Br_5_^−^)≤49.2 mol%.

For SoC≥50 % (see Figure 5, region II), the fraction of Br_2_ in Br_3_^−^ continues to decrease from x(Br_3_^−^)≈31 mol% at SoC=50 % to x(Br_3_^−^)≈9 mol % at SoC=100 %, whereas the fraction of Br_2_ in Br_5_^−^ increases up to SoC=66 % to x(Br_5_^−^)≈50 to 60 %. For SoC>66 %, the fraction of Br_2_ in Br_5_^−^ decrease as shown in Figure 5. The fraction of Br_2_, stored as heptabromide Br_7_^−^, however, increases for SoC≥50 % to x(Br_7_^−^)≈8.6 to 59.6 mol% at SoC=100 % except for [C2MIm]Br with 35.7 mol%. Overall, for SoC≥50 %, Br_2_ is increasingly stored in pentabromide and even more preferred in heptabromide. In contrast to the range SoC<50 %, there are no noticeable differences in the distribution of Br_2_ among the polybromides for the different BCAs with different alkyl side chains here. The distribution is similar for all fused salt phases with different BCAs in the range of SoC≥50 %.

#### Bromine binding strength of the BCA based on polybromide composition (SoC≤50 %)

**Figure 6:**
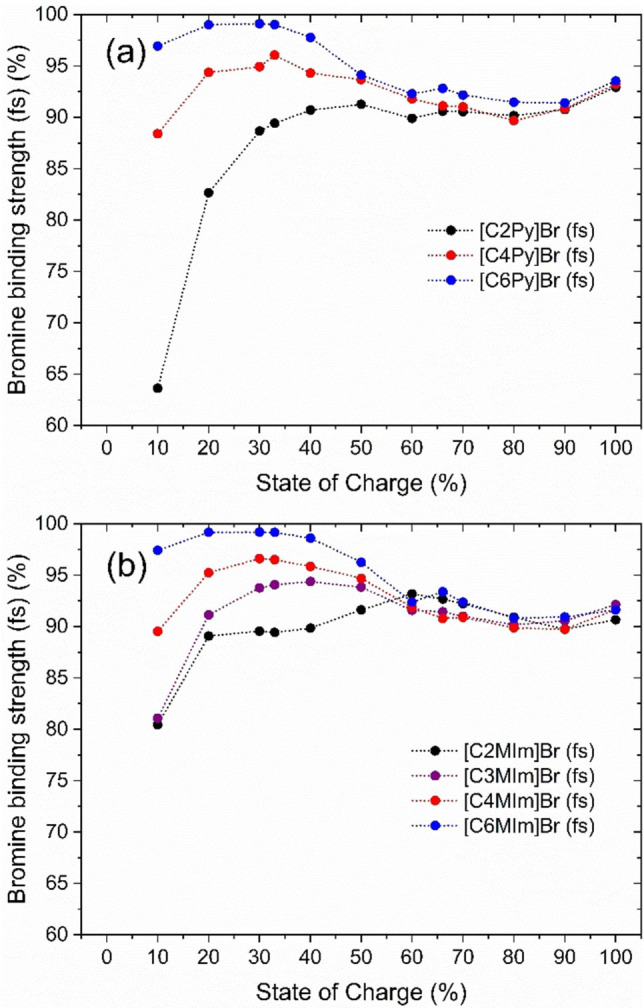
Bromine binding strength of the [BCA]^+^‐cations describing the molar fraction of Br_2_ stored in the fused salt in comparison to the absolute amount of Br_2_ in the electrolyte samples with (a) 1‐alkylpyridin‐1‐ium bromides and (b) 1‐alkyl‐3‐methylimidazol‐1‐ium bromides as BCAs at ϑ=23±1 °C.

The bromine binding strength describes how strong the Br_2_ is bound in the fused salt phase by a BCA compared to the possibility to remain in the aqueous electrolyte phase. The bromine binding strength of the BCA is defined by the molar fraction of Br_2_ stored in the fused salt phase compared to the absolute amount of Br_2_ in the sample and is shown in Figure 6.

For SoC≤50 % the binding strength depends for all investigated BCAs mainly on the alkyl side chain length and is close to 100 % for [C6Py]^+^ and [C6MIm]^+^, while a general decrease for [C4MIm]^+^, [C4Py]^+^ to [C2Py]^+^ and [C2MIm]^+^ is recognizable.

While the concentration of Br_2_ in the fused salts appears to be independent to the binding strength between the [BCA]^+^ cations and the polybromide anions Br_2n+1_^−^, the distribution of Br_2_ among the different polybromides in the fused salt is of particular importance with respect to stability of the liquid fused salts and its influence on the solubility equilibrium in Equation (1). BCAs with rising alkyl side chain length in the N‐position of [BCA]^+^ cations store Br_2_ with an increasingly higher fraction in Br_3_^−^ as shown in Figure 5 for SoC<50 %. Under this conditions, the interaction between the [BCA]^+^ cations must be stronger with Br_3_^−^ compared to Br_5_^−^ and Br_7_^−^. This interaction is intensified when *n*‐butyl or *n*‐hexyl groups are present at the BCA basic molecule components, either for pyridine or for 3‐methylimidazole. Evans et al.^[43]^ described that ammonium cations with large side chains allow polybromides to attach in their lowest energy form, which in this case would be Br_3_^−^. Therefore, the interaction of the positively charged [C6Py]^+^ and [C6MIm]^+^ cations with the negatively charged Br_3_^−^ is expected to be extremely strong, leading to a high binding strength in Figure 6. For these BCAs, hardly any Br_2_ remains in the aqueous phase and more than 98 mol% of Br_2_ is transferred to the fused salt for SoC<33 % .^[2]^ Easton et al.^[42]^ attributes this observation to the interaction between hydrogen atoms of the side chains C−H and the heteroaromatic basic structure of the BCAs with the polybromide anions, known to be stronger for Br_3_^−^.^[42]^ The dominance of Br_3_^−^ for SoC≤33 % observed in Figure 5, overlaps exactly with the strong Br_2_ binding strength shown in Figure 6 in this SoC range and leads to low concentrations of Br_2_ in the aqueous phase. As a general trend, we observed that the higher the fraction of Br_2_ in Br_3_^−^ in the fused salt the stronger the chosen [BCA]^+^ binds Br_2_ in the fused salt phase.

#### Dependence of polybromide distribution on the BCA and storage of Br_2_ in higher polybromides (SoC≥50 %)

In the SoC range of SoC≥50 % most of the [BCA]^+^ cations are transferred from the aqueous phase into the fused salt.^[2]^ More specifically, [BCA]^+^ cations are no longer detected in the aqueous phase for SoC>70 %.^[2]^ However, due to the change of the SoC the total and fused salt Br_2_ concentrations continue to rise over the SoC range. During this period, Br_2_ is absorbed at the interface into the fused salts instead of being transferred as fused salt micelle into the fused salt bulk.^[44]^ As the concentration of Br_2_ increases, lower polybromides like Br_3_^−^ and Br_5_^−^ take up Br_2_ molecules and form Br_5_^−^ and Br_7_^−^. The fractions of Br_2_ in Br_7_^−^ are rising as shown in Figure 5. The number of molecules remains constant, since each [BCA]^+^ cation is opposed by one polybromide anion to achieve charge neutrality of the solution. Although heptabromide ions are larger than tribromides, the volume per bromine molecule in heptabromide is lower than in the two polybromides Br_3_^−^ and Br_5_^−^. Br_2_ is stored compactly in higher polybromides, while the Br_2_ concentration in the fused salt and fused salt density rise.

The interaction between higher polybromides and [BCA]^+^ cations is still pronounced. However, only the presence of the [BCA]^+^ cations is decisive for this behaviour and not the length of the alkyl side chain. The general development of the bromine distribution in Figure 5 is similar for all BCAs used in the fused salt phase. Also the bromine binding strength in Figure 6 is approximately independent of the SoC and independent of the length of the alkyl side chain. Easton et al.^[42]^ described by means of DFT calculations that higher polybromides interact less strongly with the BCAs. Haller et al.^[38]^ also described a decreasing interaction between Br^−^ and Br_9_^−^ anions with [BCA]^+^ cations. This is the reason why for SoC≥50 % the binding strength is nearly independent of the BCA and its alkyl side chain when mainly Br_5_^−^/Br_7_^−^ anions are available. Similar Br_2_ concentrations in aqueous electrolyte phases,^[2]^ independent of the chosen BCA for 1‐alkylpyridin‐1‐ium and 1‐alkyl‐3‐methylimidazol‐1‐ium,^[2]^ are based on the Br_5_^−^/Br_7_^−^ composition of the fused salt.

### Conductivities of the fused salt phase

**Figure 7:**
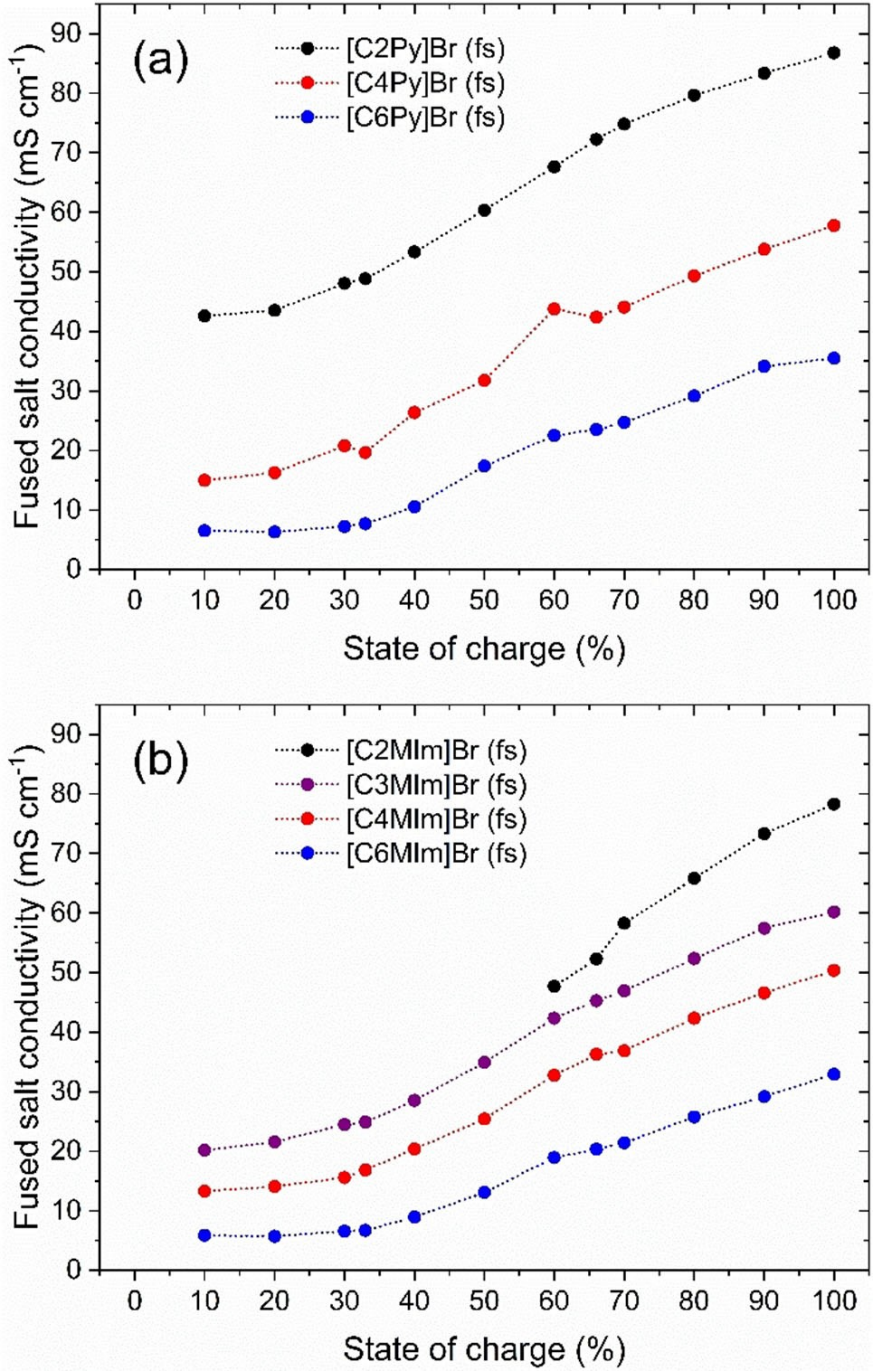
Electrolytic conductivity κ of the [BCA]Br_2n+1_ fused salts as a function of the state of charge SoC for (a) 1‐alkylpyridin‐1‐ium bromides and (b) 1‐alkyl‐3‐methylimidazol‐1‐ium bromides as BCAs with different alkyl radicals (ethyl, *n*‐propyl, *n*‐butyl and *n*‐hexyl) in the N‐position at ϑ=23±1 °C.

Since the fused salt can exist as part of an electrolyte emulsion or in the form of larger droplets and, when segregated, as a film on the electrode surface, a high conductivity of the fused salt is a prerequisite for its application in the H_2_/Br_2_‐RFB cell. The fused salts should have a high ionic conductivity in order to achieve high cell performance. The electrolytic conductivities of the evaluated fused salts are shown in Figure 7 at ϑ=23±1 °C.

In general, all equilibrated fused salt phases have conductivities (κ) between 6.89 mS cm^−1^≤κ≤89.73 mS cm^−1^ at ϑ=23±1 °C as shown in Figure 7. The reported values are significantly lower when compared to the electrolytic conductivities of their aqueous phases with conductivities in the range between 324.78≤κ≤745.00 mS cm^−1[2]^ and adding up to an increase in conductivity of 3.6 to 108 times higher than the conductivity of the fused salt. The increased conductivity of the aqueous BCA phases is mainly governed by a Grotthus‐like mechanism[^45^, ^46^] due to oxonium ions in the aqueous solution, resulting from the hydrobromic acid in the electrolyte.[^9^, ^47^] The conductivity difference between the two phases results in a large conductivity and overpotential gradient at the interface between the aqueous and fused salt in the electrolyte. Consequences for application of fused salt in the positive half‐cell are discussed in Section “Applicability of the fused salt in the H_2_/Br_2_ battery” later on in this research article.

However, in general, the ionic conductivities of the fused salts are as high as those of aqueous solutions of inorganic and organic salts. In these aqueous electrolytes, charge and mass transport in the electrical field happen by transport of the solvated ions in the electrical field due to vehicle mechanism.^[46]^ The two mechanisms differ in the way charges are transported in the electrolyte. Whereas in the vehicle mechanism oppositely charged ions migrate into opposite directions in the electric field, in the hopping mechanism a bond rearrangement occurs, for example between water and protons.^[46]^ While in the vehicle mechanism the charge is directly transported by migration of ions, charges are transported impulsively by “bond shifting” in the hopping mechanism.

As an example, conductivities of aqueous potassium chloride solutions (κ(1 M KCl)=108.62 mS cm^−1^ and κ(0.1 M KCl)=12.824 mS cm^−1^) at ϑ=25 °C^[48]^ are in the range of measured fused salt conductivities. Papancea et al.^[49]^ determined the conductivities of aqueous solutions of [C2MIm]Cl, [C4MIm]Cl and [C6MIm]Cl at ϑ=25 °C and obtained values between 5 mS cm^−1^≤κ≤85 mS cm^−1^ depending on the mass fraction of the [BCA]Cl salt. Aqueous solutions of 1.0 M 1‐ethyl‐1‐methylmorpholin‐1‐ium bromide [MEM]Br in 3.0 M ZnBr_2_ electrolytes for Zn/Br‐RFB offer conductivities of κ=44.4 mS cm^−1^ (calculated from Ref. [11]), which are in the conductivity range of the investigated fused salts. In aqueous electrolytes of ZnBr_2_/BCA supporting electrolyte salts of zinc sulphate, potassium chloride, ammonium chloride or ammonium bromide increase the conductivity up to κ=128.2 mS cm^−1^ (calculated from Ref. [11]).

Moreover, pure bromide based fused salts offer only low conductivities such as for 1‐tetrabutylammonium bromide [TBA]Br at ϑ=410 °C with κ=3.293 mS cm^−1[50]^ and [C6MIm]Br at ϑ=27 °C with κ=54.2 μS cm^−1^.^[51]^

However, in our case the polybromides present in the fused salt could be responsible of the increased conductivity compared to the reported literature values. Without considering this fact, the fused salts [MEM]Br in a Zn/Br_2_‐RFB electrolyte with 13.7≤κ≤50 mS cm^−1^ at ϑ=23 °C^[11]^ and 1‐*n*‐hexyl‐3‐methyl‐imidazol‐1‐ium nonabromide [C6MIm]Br_9_ with κ=52.1 mS cm^−1^ for ϑ=25.6 °C^[51]^ show conductivities that confirm the results of this study.

#### Dependence of the fused salt conductivity on the chosen SoC

Conductivity curves of the fused salt within the SoC range in Figure 7 show that the conductivity increases with increasing Br_2_ concentration in the electrolyte as depicted in Figure 2. This tendency is mentioned in the literature[^11^, ^12^] and is achieved from measured values, but has not been discussed in detail so far. A comparison of the conductivities κ with the polybromide composition in Figure 5 shows a strong dependence of the conductivity on the polybromide composition of the fused salt. While for small SoC values the conductivities are rather low, with Br_2_ predominantly bound in Br_3_^−^, it increases with increasing SoC if a mixture of Br_3_^−^ and Br_5_^−^ is present. For high SoCs with a mixture of Br_5_^−^ and Br_7_^−^, maximum conductivities are achieved for each [BCA]‐fused salt. There is a correlation between higher polybromides Br_2n+1_^−^ present and a conductivity increase at the same time. To investigate this phenomenon in detail, the mechanisms of the transport of charges and mass in the fused salt are investigated and discussed in the next chapter.

#### Influence of the basic molecule structure and alkyl side chain length of the BCAs on fused salt conductivity

In parallel to the distribution of polybromides in the fused salt phase, Figure 7 shows a significant difference in the conductivities for different BCAs. Based on this observation, the influence of the length of the alkyl side chain and the basic molecule structure on the conductivity is evaluated in detail. The length of the alkyl side chain in the N‐position of the BCA leads to an overall conductivity increase as seen in Figure 7. The longer the alkyl side chain in the N‐position, the lower the conductivity for each individual SoC value. This trend is observed as the hydrodynamic radius of the [BCA]^+^ cations increases with the length of the alkyl side chain. Applying the vehicle mechanism, [BCA]^+^ cations with *n*‐hexyl side chains move more slowly in the fused salt within the applied electric field due to ionic size. However, as Stegemann et al.^[52]^ and Rubinstein et al.^[53]^ suggest charge transport by means of the hopping mechanism between the polybromides, the large [BCA]^+^ cations with long alkyl side chains need to be bypassed by the hopping mechanism between the polybromide anions. At the same time, BCAs with long side chains tend to bind Br_3_^−^ more strongly and thus limit the possibilities of easy charge transport compared to equivalent polybromide mixtures of Br_3_^−^ and Br_5_^−^ (Figure 5). As the SoC increases, the conductivity and the difference between the different BCA fused salts get larger.

Conductivities of fused salts with BCAs of the same alkyl side chain, but different basic molecule structure (1‐*n*‐alkylpyridin‐1‐ium and 1‐*n*‐alkyl‐3‐methylpyridin‐1‐ium), are observed to be very similar. Slightly higher conductivities of 1‐*n*‐alkylpyridin‐1‐ium fused salts compared to 1‐*n*‐alkyl‐3‐methylimidazolium salts can be assigned to the additional methyl group in imidazolium compounds. The conductivity of the [C2MIm]Br_2n+1_ fused salt decreases more strongly and the phase forms crystals for SoC<60 %, which has been already reported in Ref. [2]. We expect that since the sizes of the evaluated BCA basic molecules (pyridine and 1‐methylimidazole) are similar, they influence the hopping mechanism in the fused salt in a similar way.

### Mechanism of charge transport and bromine mass transport in fused salt phase

Although the ionic conductivity of the fused salt is in the conductivity range of aqueous KCl solutions, we assume that the mechanism for charge and mass transport in the fused salt is not a vehicle mechanism. Instead, a charge transport by a hopping mechanism between the polybromides is suggested and discussed in detail here.

#### Comparison of existing charge and mass transport models

In aqueous solutions of KCl and [BCA]Cl or [MEM]Br/ZnBr_2_ the salts are completely or partially dissolved with water molecules. The solvation shell formed around the ions protects them against the electrostatic interactions that they can experience with other ions in the solution. In these cases, ions are able to move in the electrical field due to the mutual shielding of their charges. Charge transport and molecule transport according to the vehicle mechanism, is described by Kreuer et al.^[46]^ and strongly depends on the hydrodynamic radii of the ions. As described in the previous sections, the ions in the fused salt phase cannot experience a water solvation shell as the fused salt phase was found to be completely anhydrous. Charges of the [BCA]^+^ cations and polybromide anions Br_2n+1_^−^ cannot be shielded against their electrostatic interactions when moving in the electric field. However, since the conductivity of the fused salt phase is comparable to that of aqueous salt solutions, it is assumed that a hopping mechanism must exist between the polybromides.

**Figure 8:**
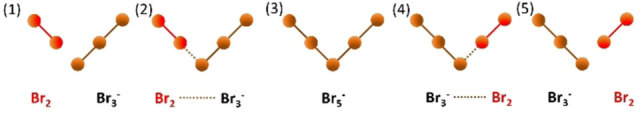
Hopping mechanism between bromine and tribromide for charge transport in fused phase according to Stegemann et al.^[52]^ and Rubinstein et al.:^[53]^ Formation of an addition bond between Br_3_^−^ and Br_2_. In step (3), Br_5_^−^ is available as transitional stage.

High conductivities in solutions and ionic liquids were studied by Stegemann et al.^[52]^ in the presence of polyhalides. Their study describes the ion conduction via a Grotthus‐like transfer of negative charge using the example of triiodide I_3_^−^ and iodine I_2_, whereby pentaiodide I_5_^−^ is formed in a transitional state and charge transport occurs by the exchange of I_2_ with the I_3_^−^ anion. This theory is additionally supported by Rubinstein et al.^[53]^ who proposed the same hopping mechanism for Br_3_^−^/Br_2_ in nitrobenzene/tetraethylammonium bromide. Charges are transferred in the solution by binding rearrangement between Br_2_ and Br_3_^−^ shown in Figure 8. Br_5_^−^ is expected to be present as a transitional stage in this model.[^13^, ^53^] However, due to the much longer addition bonds,^[13]^ pentabromide ions can bind the Br_2_ weaker than the tribromide ions.^[42]^

We assume that a hopping mechanism could be also responsible for the high conductivity of the fused salts in the bromine half‐cell electrolyte. But in contrast to Stegemann^[52]^ and Rubinstein,^[53]^ we suggest that the Br_2_ could exist only as a transitional state between two polybromides, as it has not been detected by Raman spectra.

However, we still see a significant contribution of the Br_5_^−^ from our Raman spectra (see Figure 4) that contradicts the proposed mechanism by Stegemann and Rubinstein, which assumes the presence of free Br_2_ and restricts the existence of Br_5_^−^ only as a transitional state. Based on these observations, we believe that the mechanism described in Figure 8 is not the sole responsible of the ionic movement in the investigated fused salt samples, while another hopping mechanism among polybromides could describe the high conductivity.

#### Charge and mass transport mechanism of Br_2_ by hopping mechanism for SoC<50 % within the Br_3_^−^/Br_5_^−^ polybromide regime

**Figure 9:**
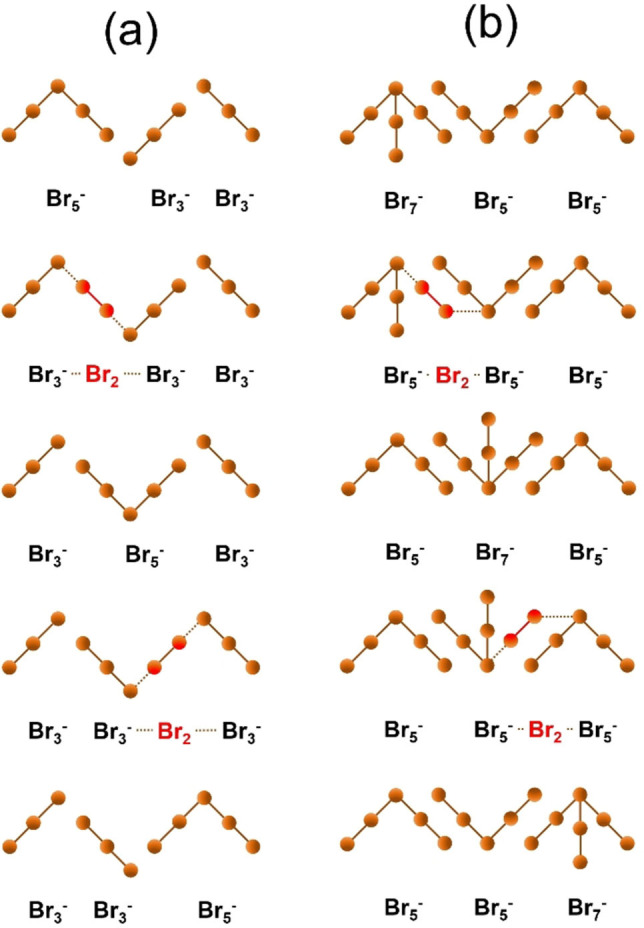
Hopping mechanism for charge transport in [BCA]Br_2n+1_ fused salts for (a) mixtures of tribromide and pentabromide and for (b) mixtures of pentabromide and heptabromide. Solid lines show the covalent bonding and the addition bonding between the bromine atoms in the polybromides. Dotted lines show the process of bonding rearrangement during the charge transport process.

As mentioned in the previous section, the conductivity of ions in the investigated fused salt phases might be governed by a hopping mechanism of the ions between the polybromides present in the electrolyte. Since the Raman spectra clearly indicated that the pentabromide is abundant in the fused salt phases for SoC<50 %, we propose a Br_2_ molecule in transition state as shown in Figure 9a instead of the initial pentabromide transition state introduced by Stegemann and Rubinstein.

Our proposed mechanism suggests that the charge transfer can occur via the formation and breakage of bonds between Br_3_^−^ and Br_5_^−^. For the mechanism to occur, the addition bond from Br_2_ to Br_5_^−^ must be weaker than the interaction of the Br_2_ and the Br_3_^−^. In this manner, the surrounding Br_3_^−^ can take up a Br_2_ molecule by forming a new addition bond, ending up on a new Br_5_^−^ cation.

In addition, the mechanism proposed in Figure 9a could be used to explain why the conductivity of the fused salt phase have been the lowest at SoC<50 %. Considering that the fused salt consists in its majority on the Br_3_^−^ anions, the smallest polybromide anions need to connect with the Br_2_ molecules of the Br_5_^−^ anions closest to their surroundings. The less Br_5_^−^ molecules are present in the fused salt, the less possible Br_2_ molecules are released from Br_5_^−^ and in consequence can be bound by Br_3_^−^, thus hindering the conductivity. As an example, for fused salts of [C6MIm]Br and [C6Py]Br at SoC=10 % more than 85 mol% of Br_2_ are bound in Br_3_^−^ while conductivities are lower than κ≤10 mS cm^−1^. Also for fused salts based on BCAs with *n*‐propyl and *n*‐butyl side chains the conductivities are lower than κ<25 mS cm^−1^ between 0<SoC≤33 %. In this range at least more than 40 mol% of Br_2_ are available in the form of Br_3_^−^. For [C2Py]Br_2n+1_ amounts of Br_3_^−^ and Br_5_^−^ are close to be equivalent and conductivities are high with 42.6≤κ≤60.4 mS cm^−1^ for SoC≤50 %.

The hypothesis of a pure fused salt consisting of only [BCA]^+^ cations and Br_3_^−^ cations would lead to even lower fused salt conductivities at room temperature. The hopping mechanism proposed in Figure 9a would not take place because no Br_2_ can be transmitted to the Br_3_^−^ due to a lack of Br_5_^−^, which is, in this situation, essential. Charge transport could take place exclusively via the vehicle mechanism, but would be strongly limited by the intense electrostatic forces of attraction and repulsion between the unsolvated ions. The formation of [BCA]Br_3_‐fused salt is the reason for sharply increasing ohmic cell resistances when fused salt is formed in the bromine half cell during the charge process as shown in Ref. [5,29].

#### Charge and mass transport mechanism of Br_2_ for SoC≥50 % within the Br_5_^−^/Br_7_^−^ polybromide regime

Increasing SoC values of SoC≥50 % lead to fused salt mixtures with mainly Br_5_^−^ and Br_7_^−^ present. More than 65 mol% of the Br_2_ are bound in Br_5_^−^ and Br_7_^−^ for SoC≥50 % and more than 80 mol% Br_2_ for SoC≥80 %. Also with increasing SoC the fused salt conductivity increases. Fabjan et al.^[12]^ mentioned that the conductivity increases with increasing Br_2_ content but did not further describe this effect. Rather, it can be assumed that higher polybromides have a key role in charge transport, with Br_2_ being exchanged. This is done by rearranging Br_2_ from a higher polybromide to a lower polybromide as depicted in Figure 9b for heptabromide and pentabromide. Although, Br_2_ transition from higher polybromides to Br_3_^−^ might additionally occur at these SoCs, we believe that the transition of Br_2_ from Br_7_^−^ to Br_5_^−^ is preferred due to lower bonding strength between Br_2_ and the Br^−^‐ion in the polybromide. The bonds between the internal bromide ion of the polybromide anion and the attached Br_2_ molecules have different length for different polybromides.^[13]^ The bond lengths between Br^−^ and Br_2_ in Br_5_^−^ and Br_7_^−^ are longer than the bond of the attached Br_2_ molecule as shown in Chen et al.^[13]^ and Br_2_ is bound weaker to the bromide ion in the polybromide. This leads to an enhanced bond breaking between the bromide of the heptabromide and an attached Br_2_ molecule, leading in the next step to a bond formation of this Br_2_ molecule in the transitional state and the pentabromide in the fused salt. The mechanism is shown in Figure 9b. The longer this bond, the easier it is to be broken. Br_2_ molecules and charges are more easily transported in Br_5_^−^/Br_7_^−^ mixtures, leading to higher electrolytic conductivities.

Haller et al.^[51]^ confirmed the possibility of a hopping‐like mechanism in these phases using the example of [C6MIm]Br_2n+1_, but assumed a charge transport to occurs between nonabromide Br_9_^−^ and Br^−^. The occurrence of Br^−^ is opposed by the fact that an ion pairing between bromide and the [BCA]^+^ cations is generally energetically unfavourable in the fused salt compared to ion pairing of Br_2_ with Br_3_^−^ or Br_5_^−^ with the [BCA]^+^ cation, as Easton et al.^[42]^ have determined on the basis of the free Gibbs energy for this process by DFT calculations on various [BCA]^+^ cations.

### Fused salt viscosity as a function of the BCA and SoC

**Figure 10:**
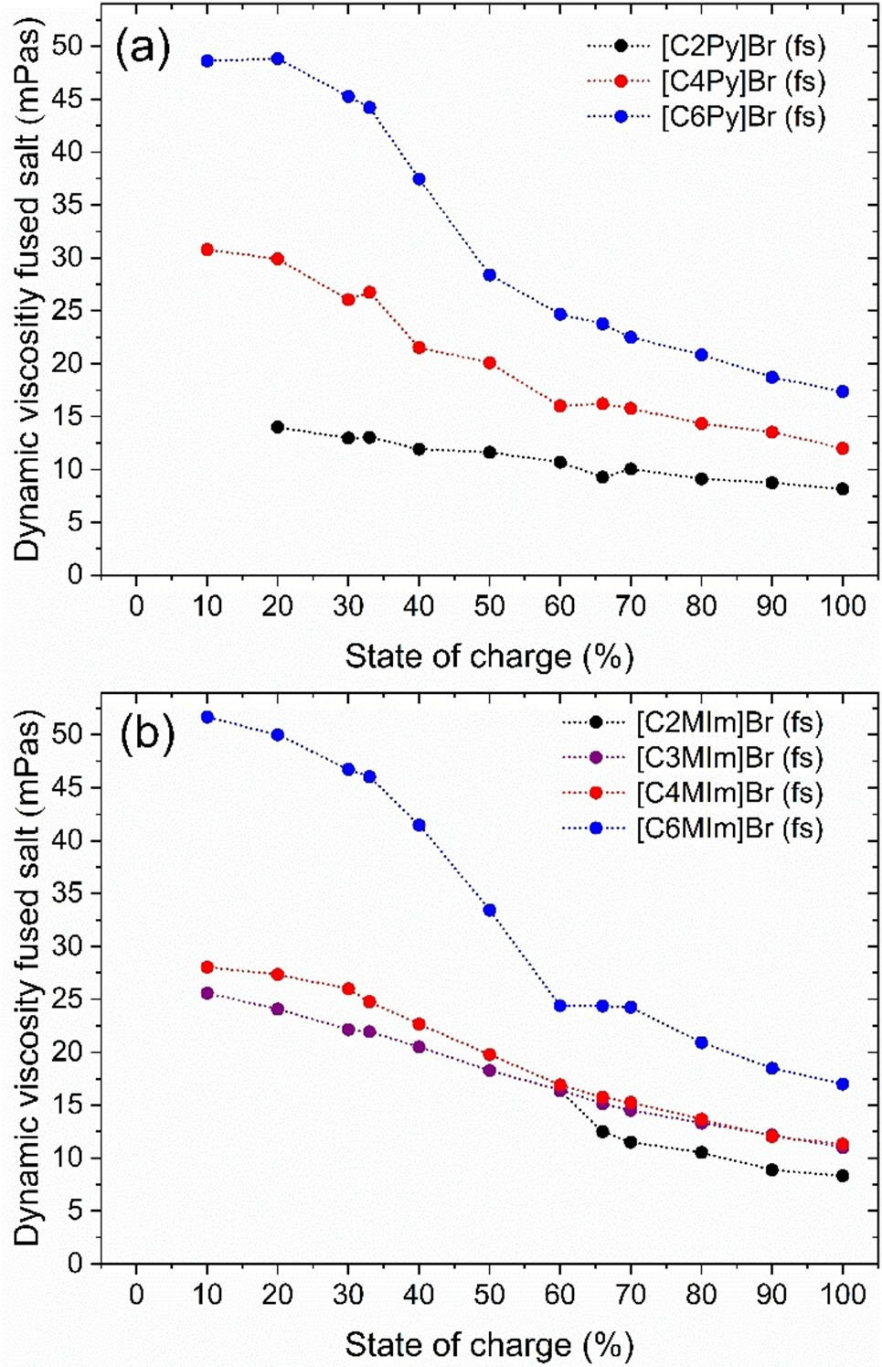
Dynamic viscosities η of the investigated fused salts based on polybromides Br_2n+1_^−^ and (a) 1‐alkylpyridin‐1‐ium and (b) 1‐alkyl‐3‐methylimidazol‐1‐ium with different alkyl side chains in N‐position of the BCA at ϑ=23±1 °C.

Additionally, the viscosity of the fused salts is a decisive parameter for the application of fused salt in the positive half‐cell of the H_2_/Br_2_‐RFB. It determines the design of the piping system and indicates whether fused salt could be used in the cell, and if so, whether this should be in a finely dispensed emulsion and in what volume ratio between fused salt and aqueous phase. Therefore, the dynamic viscosities of the fused salt phases of the individual BCAs with different alkyl side chains are determined at ϑ=23±1 °C and shown in Figure 10. The influence of the existing polybromide composition in the fused salt for the respective SoCs on the viscosity is determined and discussed.

During viscosity measurements, all fused salts showed a Newtonian flow behaviour at the investigated shear rates. A linear relationship between shear rate and force required to achieve shear of the fused salt could be determined for all fused salts and independent of the SoC. At ϑ=23±1 °C, all fused salts have dynamic viscosities (Figure 10) between 51.70≥η≥8.16 mPas and thus 8 to 51 times higher viscosities than the viscosity of water (η=1.02 mPas at ϑ=20 °C^[54]^). For viscosity measurements, [C2MIm]Br is only available in liquid aggregate state from SoC≥60 %, as already investigated and discussed in an earlier work.^[2]^

#### Influence of the BCA and alkyl side chain length on the fused salt viscosity

The dynamic viscosity depends on the selected [BCA]^+^ cation and even stronger on the length of the alkyl side chain in the N‐position. Figure 10 shows a decrease in viscosity as the SoC increases for all fused salts investigated. In general, we observed that long alkyl side chains lead to higher viscosities within the entire SoC range, following the trend: η([C6MIm]Br_2n+1_)>η([C6Py]Br_2n+1_)≫η([C4Py]Br_2n+1_)≈η([C4MIm]Br_2n+1_)>η([C3MIm]Br_2n+1_)>η([C2MIm]Br_2n+1_)>η([C2Py]Br_2n+1_). Larger [BCA]^+^ cations with larger hydrodynamic radii have a higher resistivity towards displacement, whereby Van der Waals interactions between the alkyl groups occur more frequently. The direct comparison between pyridin‐1‐ium and 3‐methylimidazol‐1‐ium derivatives does not show any fundamental differences. The length of the alkyl side chain in N‐position is decisive again. The lowest viscosity is available for fused salts of [C2Py]Br, between 8.16≤η≤14.00 mPas. The low viscosity and the stability of the liquid fused salt^[2]^ of [C2Py]Br_2n+1_ make it the most promising BCA for application in the cell in comparison to the other investigated fused salts.

#### Influence of fused salt polybromide composition on its viscosity

The viscosity change within the entire SoC range depends on the polybromide distribution in the fused salt according to Figure 10. This effect is particularly noticeable with the sharp viscosity drop observed for [C6MIm]Br, [C6Py]Br and [C4Py]Br between 30≤SoC≤60 %. The trend of decreasing viscosity coincides with the trend of decreasing Br_2_ content bound in Br_3_^−^ (see Figure 5). So it can be inferred that the higher the fraction of Br_2_ stored in tribromide, the higher the viscosity of the fused salt. Hence, if the amount of Br_3_^−^ decreases while more Br_5_^−^/Br_7_^−^ is present, the dynamic viscosity of the fused salt will decrease. As discussed before, the distribution of Br_2_ on the polybromides Br_3_^−^, Br_5_^−^ and Br_7_^−^ strongly depends on the length of the alkyl side chain length of the chosen BCA. For fused salts of [C6Py]Br and [C6MIm]Br the fraction of Br_2_ in Br_3_^−^ x(Br_3_^−^)>69 mol% for SoC≤33 %, hence their viscosities ranged among the largest measured at values ≥44 mPas. For fused salts of [C4Py]Br and [C4MIm]Br the molar fraction of 49<x(Br_3_^−^)<69 mol% for SoC≤33 % is obtained and values of viscosities are between 24.8≤η≤30.8 mPas. For [C2Py]Br an approximately similar mixture of x(Br_3_^−^)≈x(Br_5_^−^)≈50 mol% of Br_2_ bound in Br_3_^−^ and Br_5_^−^ is available, thus lower viscosity of 13 to 14 mPas is reached.

For all BCAs, the viscosity still decreases for SoC≥50 % but at a lower rate, while mixtures of Br_5_^−^/Br_7_^−^ at high SoC>50 % lead to viscosities between 8.16≤η<33.5 mPas. In general, in this SoC range, the fraction of Br_2_ in Br_3_^−^ is x(Br_3_^−^)≤35 mol%, with values nearly independent of the BCA and its alkyl side chain length. Whereas, x(Br_7_^−^) rises from around 10 mol% to around 50 mol%, while Br_5_^−^ contains around 31 to 60 mol % Br_2._ The observation that the viscosity decreases for higher polybromides can be explained by the following theory.

If one considers that the fused salt is composed on the one hand by single positively charged organic [BCA]^+^ cations and on the other hand by different but single negatively charged polybromide anions Br_3_^−^, Br_5_^−^ and Br_7_^−^, both ions are not shielded versus electrostatic interactions between the ions due to the lack of a solvation shell. The displacement of individual ions against each other is thus subject to resistivity. While oppositely charged ions cannot be shifted by attractive forces, ions with the same charge are repelled by repulsive forces. In Br_7_^−^, the charge density is lower than in Br_3_^−^, due to its larger hydrodynamic radius. The interactions between the polybromides and the individual [BCA]^+^ cations strongly depend on both species and are weaker for higher polybromides like Br_5_^−^ and Br_7_^−^, compared to strongly interacting Br_3_^−^. Haller et al.^[51]^ determined on the basis of ^1^H NMR analysis that nonabromide Br_9_^−^ enters into much weaker interactions with [C6MIm]^+^ than bromide Br^−^. Easton et al.^[42]^ also calculated that there is a strong ion pairing between Br_3_^−^ and [BCA]^+^ ions, while the tendency for ion pairing is weaker between Br_5_^−^ and [BCA]^+^. The addition of Br_2_ with rising SoC leads to the formation of higher polybromides from Br^−^ to Br_7_^−^. For the investigated fused salts, the polybromides interact less strongly with the [BCA]^+^ cation with increasing Br_2_ loading. They can be moved in the fused salt more easily by an external force, which allows a higher shear rate. In this case, BCAs with longer alkyl side groups tend to interact very strongly with tribromide Br_3_^−^ leading to high viscosities in the fused salt.

### Temperature stability of the electrolyte exemplified by [C2Py]Br as BCA

**Figure 11:**
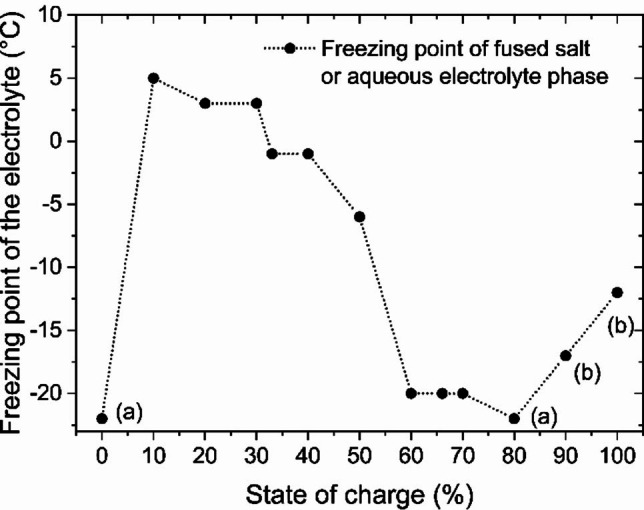
Freezing temperatures of HBr/Br_2_/H_2_O electrolytes with [C2Py]Br as BCA in dependence on the SoC. All freezing temperatures without alphabetical labelling describe that at this temperature and below the fused salt phase is completely or partially crystallized. Points labelled with (a) describe samples at SoC=0 and 80 % which form stable liquids in both phases to at least ϑ=−22 °C. Points labelled with (b) at SoC=90 and 100 % show electrolytes in which fused salt phases remain liquid below the indicated temperatures, but the aqueous phase crystallizes.

Since [C2Py]Br emerges as a potentially applicable BCA for the application of fused salt due to its high ionic conductivity and low viscosity compared to the other BCAs investigated, the temperature stability of the fused salt phase is examined here as an example. For an application of H_2_/Br_2_‐RFB in many countries worldwide, temperatures far below ϑ=0 °C can be reached in winter. Both electrolyte phases, the aqueous and the fused salt phases, must remain as a liquid within the entire SoC range. Therefore, electrolyte samples consisting of an aqueous electrolyte phase and a fused salt are cooled down from room temperature starting from ϑ=23±1 °C. The freezing temperatures of the electrolyte are shown in Figure 11.

When the electrolytes are cooled down, safe use of the two‐phase liquid electrolyte can be observed at a temperature ϑ>5 °C within the whole SoC range. For ϑ>5 °C, electrolytes of [C2Py]Br as BCA in the considered mixture can be used in the cell for cycling.

For SoC=0 %, the electrolyte consists of a bromine‐free mixture of 7.7 M HBr in H_2_O and 1.11 M [C2Py]Br and can be cooled down to at least ϑ=−22 °C without crystallisation. Due to the BCA content and especially the high content of HBr, there is a melting point depression in the mixture of predominantly HBr and H_2_O. In the electrolyte samples for SoC>0 %, a two‐phase electrolyte mixture is then present, whereby each phase can crystalize individually when temperature is reduced.

Between 10≤SoC≤70 %, crystallization of the fused salt phase occurs with decreasing temperature as shown in Figure 11. The fused salt phase is solid and can no longer be pumped or moved. The freezing temperature of the fused salt phase decreases from SoC=10 % with ϑ=5 °C to SoC=70 % with ϑ=−20 °C. For higher SoCs, it is possible to use the electrolyte at temperatures below ϑ=0 °C in the H_2_/Br_2_ RFB. For SoC≥80 %, the fused salt phase remains liquid in the electrolyte at least down to a temperature of ϑ=−22 °C. For crystallization, it is shown that the present polybromides in the fused salt phase have an influence on crystal formation with decreasing temperature. Although the influence of temperature on the distribution of bromine in the polybromides has not been investigated, based on the Br_2_ distribution at ϑ=23±1 °C, a clear influence of the polybromides on the freezing temperature can be observed. The decrease in freezing temperature roughly parallels the decrease in Br_3_^−^ concentration in the fused salt phase from x(Br_3_^−^)=48 % at SoC=20 % to x(Br_3_^−^)=8 % at SoC=100 %. As known in Küttinger et al.,^[2]^ crystallization of positively charged [BCA]^+^ cations occurs by formation of tribromide salts [BCA]Br_3_. This has been explained in detail using the example of [C2MIm]Br_3_ in Ref. [2] For 10≤SoC≤50 % with a 30≤x(Br_3_^−^)≤48 %, the fraction of Br_3_^−^ and Br_5_^−^ is predominant. Due to the smaller hydrodynamic radius and the higher charge density of Br_3_^−^, crystallization occurs preferentially and is therefore possible at temperatures between 0≤ϑ≤5 °C. As explained in detail in Ref. [42] a preferential aggregation of tribromide with the BCAs occurs. For SoC>50 %, fractions of Br_5_^−^ and Br_7_^−^ predominate in the mixture. In these mixtures, crystallization also occurs, but at much lower temperatures than at lower SoCs. Br_5_^−^ and Br_7_^−^ have a larger hydrodynamic radius and a lower charge density compared to Br_3_^−^. Hence, they are less likely to bond with the [BCA]^+^ cations or to crystallize only at lower temperatures.

At SoC=90 and 100 %, the aqueous electrolyte phase crystallizes at ϑ=−17 °C and−12 °C, respectively. In the aqueous phase, the solutions contain a bromine concentration of around 0.26 M^[2]^ and HBr with concentration between 1.00 M and 1.67 M. Overall, the sum of HBr and Br_2_ concentration is lowest at this point. The melting point depression in the aqueous phase is lowered by low HBr concentrations, so that crystallization of the aqueous phase for both samples occurs above ϑ=−22 °C.

### Applicability of the fused salt in the H_2_/Br_2_ battery

The application of the fused salt is advantageous for the discharge process due to bromine concentrations c(Br_2_, fs)>4 M, since high discharge current densities based on high bromine concentrations can be expected. However, the conductivity of the fused salt compared to the aqueous electrolyte solution in electrolytes is not sufficient for the application in H_2_/Br_2_‐RFB. For the application, there is an explicit difference between the conductivities of the aqueous phase with κ(aq)>298 mS cm^−1^ and the fused salt with κ(fs)<89.7 mS cm^−1^, which increases with decreasing SoC as shown in Figure 7 and in conductivity values in Ref. [2]. Thus, the feasibility of obtaining high current densities is limited by the low conductivity of the fused salt compared to the one of the aqueous phase.

**Figure 12:**
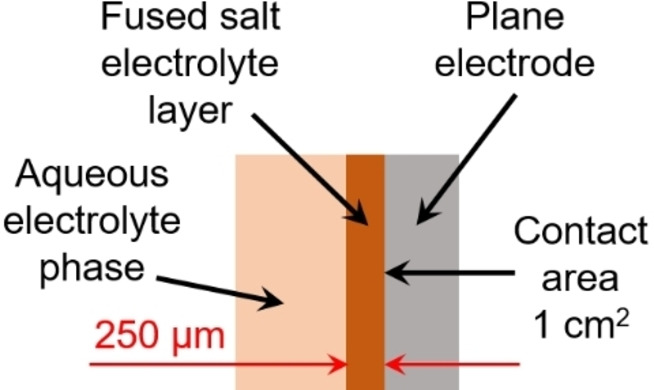
Scheme of a fused salt layer with a constant thickness of 250 μm in front of the electrode surface to illustrate the calculation of conductivity overpotentials occurring in this layer.

A comparison of the possible voltage losses between the aqueous phase and the fused salt phase clearly shows the differences between the two electrolytes. The ohmic overpotential arising from the BCA‐containing electrolytes can be calculated using the phase conductivities determined in this study and in Ref. [2]. As an example, the values are estimated for the [C_X_Py]Br_2n+1_ BCA series and are summarized in Table 2. For the calculation, it is assumed that the selected electrolyte phase is present in front of the electrode surface with a layer thickness of 250 μm, and that the cell is charged or discharged applying a current density of 100 mA cm^−2^ (as seen in Figure 12).

**Table 2:** Ohmic overpotential based on the conductivity of fused salts and aqueous electrolytes with [C2Py]Br, [C4Py]Br and [C6Py]Br in an electrolyte film of 250 μm thickness and a current density of 100 mA cm^−2^.

Chosen [BCA]Br	[C_X_Py]Br_2n+1_ fused salt		[C_X_Py]Br_2n+1_ aqueous phase	
	SoC 20 %	SoC 90 %	SoC 50 % (κ _max_)	SoC 100 % (κ _min_)
[C2Py]Br	57.4 mV	29.9 mV	3.51 mV	7.46 mV
[C4Py]Br	153.7 mV	46.5 mV	3.43 mV	7.70 mV
[C6Py]Br	395.4 mV	73.2 mV	3.36 mV	7.45 mV

In a comparison to the aqueous electrolyte, the results in Table 2, clearly suggest that the ohmic drop resulting from the fused salt phase is much higher than its equivalent in the aqueous phase. The ohmic overpotential caused by the fused salt changed depending on the SoC and the [BCA]^+^ cation and it is a consequence of the phase low conductivity. Contrarily, the aqueous electrolyte solutions of the same layer thickness show much lower overpotentials in the single‐digit mV range, as also shown in Table 2.

This factor distinguishes the H_2_/Br_2_‐RFB from the Zn/Br_2_‐RFB, since small current densities are used in Zn/Br_2_‐RFB with the aim of uniform zinc deposition free of dendrites and the conductivities of the aqueous Zn/Br_2_‐RFB electrolytes and the fused salts are similar as can be seen in the example of Eustace:^[11]^ Aqueous phase: 44.4 mS cm^−1^≤κ≤128.2 mS cm^−1^ and fused salt: 13.7 mS cm^−1^≤κ≤50 mS cm^−1^.

An area‐covering layer of the fused salt phase on the electrode surface is not practical for the application in H_2_/Br_2_‐RFB. Ultimately, the application of fused salt thus also strongly depends on the configuration of the electrode. While dense felt electrodes offer a high specific surface for the reaction, they also offer an increased possibility of adhesion of fused salt to their surface.

Furthermore, for practical application, pumping a pure fused salt is not appropriate. Since the energy required for pumping increases proportional to the dynamic viscosity, the energy consumption would be 8 to 51 times higher than for water. The large variation of the fused salt viscosity in addition influences the choice of pumps. As an alternative to reduce the flow resistance, the fused salt could be transported in the form of drops within the aqueous phase for example in an emulsion, without touching the walls of the battery supply tubes. However, in any case, discharging the pure fused salt in the cell would not be possible if there was no aqueous phase to absorb the protons.

For the application of fused salt in H_2_/Br_2_‐RFB, it is necessary to investigate the following points in future studies, while taking the properties of the fused salt into account: Structure of the electrode in the positive half cell (felt, carbon paper, thickness of the cell, retention time characteristics), adhesion of the fused salt to the electrode surface as a function of the SoC, coalescence characteristics of emulsions as a function of the fused salt composition, and the bromine/bromide redox kinetics from the fused salt phase.

## Conclusion

In aqueous HBr/Br_2_ electrolytes for H_2_/Br_2_‐RFB, bromine complexing additives are applied to bind volatile bromine in a separate and second liquid phase; called fused salt, in the electrolyte. In this work, the chemical and operational relevant properties of these fused salts have been investigated. The properties of fused salts of seven different BCAs from the group of 1‐alkylpyridin‐1‐ium bromides and 1‐alkyl‐3‐methylimidazol‐1‐ium bromides with the alkyl side chains ethyl, *n*‐propyl, *n*‐butyl and *n*‐hexyl have been determined:

All BCAs form a separated fused salt phase in contact with polybromides Br_3_^−^, Br_5_^−^ and Br_7_^−^, with only [C2MIm]Br as BCA tending to crystallize the fused salt phase. The previously widely unverified composition of fused salt phases has been determined for the first time and consists of anhydrous and bromine‐free ionic liquids composed purely of [BCA]^+^ cations, and the polybromide anions Br_3_^−^, Br_5_^−^ and Br_7_^−^. It is a pure ionic liquid for all chosen BCAs and the whole investigated SoC range of the electrolyte.

Bromine is stored in concentrations between 4.98≤c(Br_2_, fs)≤13.62 M in the fused salt, resulting in capacities between 267 to 730 Ah L^−1^, which are largest known for RFBs electrolytes. However, operation of the electrolyte is only feasible in combination with the aqueous electrolyte phase in the bromine half‐cell of the RFB.

With conductivities between 7≤κ(fs)≤90 mS cm^−1^, the fused salt phases are about 3.6 to 108 times less conductive than their corresponding aqueous electrolyte solutions. Simultaneously, their conductivities are comparable to the ones of aqueous KCl solutions with c(KCl)<1 M. This indicates an exceptionally high conductivity of the fused salt phase at room temperature, despite the fact that it is a pure fused salt. The transfer of charges and Br_2_ in the fused salt phase takes place by a hopping mechanism comparable to the Grotthus‐mechanism between water and protons. Here a Br_2_ molecule is transferred from a higher polybromide such as Br_5_^−^/Br_7_^−^ to a lower polybromide Br_3_^−^/Br_5_^−^. Lower polybromides bind Br_2_ more strongly by forming an addition bonding than higher polybromides. Charge is transported through the electrolyte by the release and replacement of these bonds. It is proposed that Br_2_ is present as a transition state in the hopping mechanism, as it was not detected in the fused salt phases. Hence, original models by Stegemann^[52]^ and Rubinstein,^[53]^ which assume free Br_2_ molecules in the fused salt, do not apply to describe the conductivity of the fused salt phases in this work. In the Raman spectra of the sample only Raman peaks of Br_3_^−^, Br_5_^−^ and Br_7_^−^ polybromides are registered, while pure Br_2_ is not available in the fused salt to fulfil the mechanism of Stegemann and Rubinstein.

The viscosities of the fused salt phase between 8<η<52 mPas strongly depend on the BCA and on the polybomides present, whereby BCAs with short alkyl side chains are to be preferred with high amounts of Br_5_^−^ and Br_7_^−^.

An application of the fused salt in combination with the aqueous electrolyte phase is not advantageous by directly pumping the fused salt into the cell due to low conductivity and high viscosity. For Zn/Br_2_‐RFB instead, or in form of emulsions, an application could be possible, expecting adjusted bromine half‐cell design. The most promising electrolytes are those based on 1‐ethylpyridin‐1‐ium bromide [C2Py]Br as BCA, since its fused salt reaches a maximum volumetric capacity and at the same time has the highest conductivities and lowest viscosities. However, Zn/Br_2_‐RFB electrolytes are of different composition including zinc bromide (ZnBr_2_) or conducting salts like ammonium bromide (NH_4_Br) and a general transformation of this work to Zn/Br_2_‐RFB electrolytes is not feasible. Further investigations for [C2Py]Br fused salts are necessary in the form of aqueous/fused salt emulsions in thin bromine electrodes with carbon paper for use in H_2_/Br_2_‐RFB.

## Experimental Section

A detailed description of the testing methods is given in the Supporting Information (chapter 1) for this article to enable reproducibility of the measurements. An abbreviated form is reproduced here.

### Reagents and synthesis of bromine complexing agents

Bromine electrolyte samples consist of hydrobromic acid, bromine and distilled water. All used BCAs (Table 1) are synthesized from 1‐methylimidazole, pyridine, bromoethane, 1‐bromopropane, 1‐bromobutane and 1‐bromohexane. Suppliers and purity of chemicals are listed in the Supporting Information. Investigated BCAs are listed with name and structure in Table 1. BCAs are synthesized in a one step and solvent‐free nucleophilic substitution reaction (S_N_2 preferred) according to Dzyuba et al.^[55]^ Detailed information on their synthesis and cleaning process are described in Ref. [2]. ^1^H NMR and ^13^C NMR spectra of the synthesized substances are recorded and are consistent with NMR shifts found in the literature.^[56]^

### Electrolyte formulation

Fused salt properties are investigated ex situ from the fused salt phases of the electrolyte mixtures for the synthesized BCAs. Electrolyte samples for SoC 10, 20, 30, 33, 40, 50, 60, 66, 70, 80, 90 and 100 % are prepared with a total volume of 30 mL each, while concentrations in dependence of the SoC are shown in Table S1 in the Supporting Information. The SoC range is defined for SoC=0 % with 7.7 M HBr, 1.11 M [BCA]Br and 0 M Br_2_ in aqueous solution and for SoC=100 % with 1 M HBr, 1.11 M [BCA]Br and 3.35 M Br_2_ in the aqueous solution. SoC definition is derived from an earlier study investigating the limits in the cycling capacity of pure HBr/Br_2_/H_2_O electrolytes.^[9]^ The electrolyte separates into two phases. The choice of the BCA concentration of 1.11 M [BCA]Br for all samples bases on the expectation to store theoretically 3 molecules of Br_2_ at one [BCA]^+^ cation as heptabromide at SoC=100 % and is further explained in Refs. [5,44].

### Fused salt bromine concentration

Concentrations of Br_2_ in the fused salt phase of the seven investigated BCAs are determined for all chosen SoCs. Br_2_ concentrations of fused salt are calculated by measuring the volume of the fused salt V(fs), the aqueous phase V(aq), utilization values of measured Br_2_ concentration of aqueous phase c(Br_2_, aq) for each sample and the total mass of Br_2_ (m(Br_2_, total)) in the sample. The bromine concentration is calculated by the following Equation 2:

(2) $$c\left({Br}_{2},fs\right)=\;\frac{m({Br}_{2},\;total)}{M\left({Br}_{2}\right)\cdot V\left(fs\right)}-\frac{c\left({Br}_{2},aq\right)\cdot V\left(aq\right)}{V\left(fs\right)}$$

M(Br_2_) is the molar mass of Br_2_. The total mass of Br_2_ depends on the SoC and is shown in Table S2 in the Supporting Information. The concentrations of Br_2_ in the aqueous phase are determined experimentally. The method is described in the Supporting Information of this article and in detail in Ref. [2]. The values for c(Br_2_, aq) are tabulated in the Supporting Information of Ref. [2].

### Fused salt density

The density of the fused salt samples is investigated at ϑ=23±1 °C by means of a pipette. Electrolyte samples are positioned on a balance, the pipette tip is dipped through the aqueous electrolyte into the fused salt phase and a volume of ΔV(fs)=1 mL of fused salt is sampled. The pipette is pulled out of the electrolyte and the change in mass is the fused salt mass Δm(fs) corresponding to ΔV(fs)=1 mL. Density of the fused salt is calculated from Δm(fs) and ΔV(fs) (Supporting Information).

### Concentration of [BCA]^+^ cations in fused salt phases

Concentrations of [BCA]^+^ cations in the fused salt are calculated from the volumes of the fused salt phase V(fs) and the aqueous electrolyte phase V(aq), the total [BCA]^+^ concentration of each sample (1.11 M) and the [BCA]^+^ concentrations of the aqueous electrolyte phase following Equation 3:

(3) $$c\left({\left[BCA\right]}^{+},\;fs\right)=\frac{1.11\;\;M\;\cdot 0.03\;\;L}{V\left(fs\right)}-\frac{c\left({\left[BCA\right]}^{+},\;aq\right)\cdot V\left(aq\right)}{V\left(fs\right)}$$

The values of c([BCA]^+^, aq) are tabulated in Ref. [2] and applied in Equation (3) to calculate the [BCA]^+^ concentrations in the fused salt. Concentrations of [BCA]^+^ cations in aqueous electrolyte phase c([BCA]^+^, aq) are determined by Raman spectroscopy. The method is described in detail in Ref. [2].

### Polybromide determination and Br_2_ distribution on the polybromides in fused salt phases

By means of Raman spectroscopy on the fused salt phase samples, the occurrence of the different polybromides Br_3_^−^, Br_5_^−^ and Br_7_^−^ in fused salt series of the [BCA]^+^ cations at different SoCs is investigated. The procedure, parameter and equipment are described in the Supporting Information. Peak areas of the following stretching oscillations of the polybromides are determined by fitting according to the Lorentz model and using the iteration algorithm according to Levenberg‐Marquardt: $\tilde{\nu }$ (Br_3_^−^, sym.)≈164–170 cm^−1^,[^13^, ^41^, ^42^] $\tilde{\nu }$ (Br_3_^−^, antisym.)≈190‐198 cm^−1^,[^13^, ^37^, ^42^] $\tilde{\nu }$ (Br_5_^−^, antisym.)≈210 cm^−1^,[^13^, ^42^] $\tilde{\nu }$ (Br_5_^−^, sym.)≈253–255 cm^−1^ [^13^, ^37^, ^38^, ^39^, ^42^], $\tilde{\nu }$ (Br_7_^−^, sym.)≈269 cm^−1^.[^13^, ^38^] The fraction of the area of a symmetrical stretching oscillation in relation to the sum of all areas of the symmetrical stretching oscillations of Br_3_^−^, Br_5_^−^ and Br_7_^−^ gives the fraction of the Br_2_ concentration present in each polybromide x(Br_3_^−^), x(Br_5_^−^) and x(Br_7_^−^). A detailed description and explanation of the method shown in another work of the authors.^[9]^ All Raman spectra of the aqueous phase are shown in Figure S1–S7 in the Supporting Information.

### Electrolytic conductivities of fused salt posolyte

Ionic conductivities of the fused salt phases of all chosen BCAs are determined at various SoCs at ϑ=23±1 °C. In a conductivity cell ohmic electrolyte resistances R_ELECTROLYTE(measurement)_ are measured by help of potentiostatic impedance spectroscopy. The electrolyte conductivity κ _ELECTROLYTE(fs)_ is calculated taking the cell constants K_Conductometer_ into account. Equations for calculation and measurement of K_Conductometer_ are described in the Supporting Information of this article in detail.

### Rheological behaviour of fused salt phase

Rheological data of liquid fused salts are measured at various SoC using a plate/plate configuration rheometer. Shear rates of 10 up to 100 s^−1^ at ϑ=23±1 °C with a distance of 0.5 mm between the plates are applied in the measurements. Dynamic shear viscosities η are calculated. Details on the instrument are mentioned in the Supporting Information.

### Temperature stability of the two phase electrolyte

Electrolytes with [C2Py]Br as BCA at different SoCs are cooled down in a climatic chamber from ϑ=25 °C in steps of Δϑ=−1 °C up to a maximum of ϑ=−22 °C and visually examined whether and which of the two electrolyte phases crystallizes.

## Credit author statement

Michael Küttinger: Conceptualization, Methodology, Experimental investigation, Data analysis, Visualisation, Writing – Original Draft, Writing‐Editing and Review, Supervision of master students. Paulette A. Loichet Torres: Experimental investigation, Data analysis, Writing‐Editing and Review. Emeline Meyer: Synthesis of substances, Experimental investigation. Peter Fischer: Supervision, Resources.

## Conflict of interest

The authors declare no conflict of interest.

## Supporting information

As a service to our authors and readers, this journal provides supporting information supplied by the authors. Such materials are peer reviewed and may be re‐organized for online delivery, but are not copy‐edited or typeset. Technical support issues arising from supporting information (other than missing files) should be addressed to the authors.

Supporting Information
