# Supplementary material for: Properties of Bromine Fused Salts Based on Quaternary Ammonium Molecules and Their Relevance for Use in a Hydrogen Bromine Redox Flow Battery
Source: Chemistry. 2022 Feb 18;28(13):e202103491. doi: 10.1002/chem.202103491 (PMC9304276; doi:10.1002/chem.202103491)
Supplement: Supplementary file 1 — Supporting Information [file CHEM-28-0-s001.pdf]

Chemistry–A European Journal

Supporting Information

Properties of Bromine Fused Salts Based on Quaternary Ammonium Molecules and Their Relevance for Use in a Hydrogen Bromine Redox Flow Battery

Michael Küttinger,* Paulette A. Loichet Torres, Emeline Meyer, and Peter Fischer

In the ESI the reader finds detailed information on the applied measurement methods, parameter settings for measurements, chemicals and purities as well as evaluation steps. In addition, individual results can be found in the form of graphs and tables starting from chapter 2 of the ESI. These data supplement the manuscript and ensure completeness of the work, as not all measurement results can be included in the manuscript for reasons of clarity.

1. Detailed description of experiments and methods

In addition to the "Experimental Section" of the manuscript, experiments and methods are described in detail in this chapter of the ESI in order to enable reproducibility of the results. Experiments and evaluation methods are described in detail. Utilized chemicals are named including their manufacturer and purity. For other materials names and manufacturers are given.

1.1. Reagents for electrolyte preparation

The used reagents for preparation of bromine electrolyte samples for this fused salt phase study are hydrobromic acid (48% w/w, Alpha Aesar), bromine (≥99%, Sigma Aldrich) and distilled water (PURELAB Ultra Analytic/Veolia-ELGA). All used BCAs are synthesized from 1-methylimidazole (99%, Aldrich), pyridine (99%, Alpha Aesar), bromoethane (98%, Alpha Aesar), 1-bromopropane (99%, Alpha Aesar), 1-bromobutane (98%+, Alpha Aesar) and 1-bromohexane (98%, Alpha Aesar).

1.2. Synthesis of bromine complexing agents

Investigated BCAs are listed in Table 1 of the research article, including chemical structure, name and abbreviation. All used BCAs ([C2Py]Br, [C4Py]Br, [C6Py]Br, [C2MIm]Br, [C3MIm]Br, [C4MIm]Br, [C6MIm]Br), are synthesized in a one step and solvent-free nucleophilic substitution reaction (S_N2 preferred) according to Dzyuba et al. [1] by alkylation reaction [2,3]. Tertiary amines and bromoalkanes exclusively participate in the reaction. Characteristic colours of BCA crystals and detailed information on their synthesis and cleaning process are described in ref. [4]. 1.5 mol of each [BCA]Br are produced. ^{1}H NMR and ^{13}C NMR spectra of the synthesized substances are recorded and confirm the absence of any educts as well as water left in the product. ^{1}H NMR and ^{13}C NMR shifts, as well as instrument data are presented in chapter 2 in this ESI. They are adapted from our ref. [4] and are consistent with NMR shifts found in the literature [3,5–8].

1.3. Electrolyte formulation

Fused salt properties are investigated ex situ from the fused salt phases of aqueous/fused salt electrolyte mixtures. For the synthesized BCAs [C2Py]Br, [C4Py]Br, [C6Py]Br, [C2MIm]Br, [C3MIm]Br, [C4MIm]Br and [C6MIm]Br samples are prepared for different SoCs. The different SoCs represent different concentration mixtures of the electrolytes and are defined for SoC = 0 % with 7.7 M HBr, 1.11 M [BCA]Br and 0 M Br_2 in aqueous solution and for SoC = 100 % with 1 M HBr, 1.11 M [BCA]Br and 3.35 M Br_2 in the aqueous solution. SoC definition is derived from an earlier study investigating the limits in the cycling capacity of pure $HBr/Br_2/H_2O$ electrolytes [9]. Electrolyte samples for SoC 10, 20, 30, 33, 40, 50, 60, 66, 70, 80, 90 and 100 % are prepared with a total volume of 30 mL each and concentrations in dependence of the SoC are shown in Table S1:

Table S1: Total concentrations of Br_2, HBr and [BCA]Br in mol L^{-1} in investigated electrolyte samples at chosen SoC for ex situ electrolyte investigation.

	Total concentrations of Br_2, HBr and [BCA]Br in mol L^{-1} in investigated electrolyte samples at chosen SoC												
SoC / %	0	10	20	30	33	40	50	60	66	70	80	90	100

c(HBr) / M	7.70	7.03	6.36	5.69	5.49	5.02	4.35	3.68	3.28	3.01	2.34	1.67	1.00
c([BCA]Br) / M	1.11	1.11	1.11	1.11	1.11	1.11	1.11	1.11	1.11	1.11	1.11	1.11	1.11
c(Br_2) / M	0.00	0.34	0.67	1.01	1.11	1.34	1.68	2.01	2.21	2.35	2.68	3.02	3.35

The choice of the BCA concentration of 1.11 M [BCA]Br for all samples is based on the expectation to store theoretically 3 molecules of Br_2 with one $[BCA]^+$ cation as Br_7^- at SoC 100 %. For SoC 33 % 1 molecule of Br_2 would be stored theoretically as Br_3^- for each $[BCA]^+$ cation and at SoC 66 % 2 molecules of Br_2 should be stored for each $[BCA]^+$ cation as Br_5^-. The amount of water is kept constant for all samples and bases on a 7.7 M HBr solution defined for SoC 0 % to maintain the theoretical amount of water in the catholyte during cell operation. For two weeks, electrolyte samples are shaken daily once to achieve a chemical equilibrium of the ingriedients between the two electrolyte phases. The procedure is equivalent to the one of an earlier study focusing on the characterization of the aqueous phase of the same electrolytes from ref. [4].

1.4. Fused salt density

The densitiy of the fused salt samples of all 7 BCAs for the different SoCs (10, 20, 30, 33, 40, 50, 60, 66, 70, 80, 90 and 100 %) at ϑ = 23 ± 1 °C is investigated with a ELEK SE4 STD 1-10ML pipette. The samples are positioned on a balance, the pipette tip is dipped through the aqueous electrolyte into the fused salt phase and a volume of ΔV(fs) = 1 mL of fused salt is sampled. The pipette is pulled out of the electrolyte and drops adhering to the outside are drifted off at the edge of the sample. The change in mass Δm(fs) of the electrolyte sample corresponds to the mass of the fused salt Δm(fs) in the pipette. The density of the fused salt is calculated by Eqn. 1:

Eqn. 1 $$\rho(\mathrm{fs}) = \frac{\Delta \mathrm{m(fs)}}{\Delta \mathrm{V(fs)}}$$

1.5. Fused salt bromine concentration

Concentrations of Br_2 in the fused salt phase of the seven investigated BCAs ([C2Py]Br, [C4Py]Br, [C6Py]Br, [C2MIm]Br, [C3MIm]Br, [C4MIm]Br and [C6MIm]Br) are determined and all chosen SoCs (10, 20, 30, 33, 40, 50, 66, 70, 80, 90 and 100 %). Br_2 concentrations of fused salt are calculated by measuring the volume of both, the fused salt V(fs) and the aqueous phase V(aq), while utilization values of measured Br_2 concentration of aqueous phase for each sample and the total amount or total mass of Br_2 in the sample. The bromine concentration is calculated by the following Eqn. 2:

Eqn. 2 $$\mathrm{c(Br_2, fs)} = \frac{\mathrm{m(Br_2,total)}}{\mathrm{M(Br_2)\cdot V(fs)}} - \frac{\mathrm{c(Br_2,aq)\cdot V(aq)}}{\mathrm{V(fs)}} \qquad [\mathrm{c(Br_2, fs)}] = \mathrm{mol\ L^{-1}}$$

(c(Br_2, fs) = concentration of Br_2 in fused salt in mol L^{-1},m(Br_2, total) = total mass of Br_2 in sample in g, M(Br_2) = molar mass of Br_2 (159.8 g mol^{-1})). Volumes of the phase are measured volumetrically with a precision of +/- 0.25 mL.The total mass of Br_2 depends on the SoC and is shown in Table S2:

Table S2: Total mass of Br_2 used in the investigated samples depending on the SoC. The total amount of Br_2 is distributed in the sample between the aqueous electrolyte and fused salt phase.

SoC / %	10	20	30	33	40	50	60	66	70	80	90	100

m(Br_2, total) / g	0.54	1.07	1.61	1.77	2.14	2.68	3.21	3.52	3.75	4.28	4.82	5.35

The concentrations of Br_2 in the aqueous phase are determined experimentally by linear chronoamperometry at a rotating disk electrode at a rotation speed (ω) of ω = 1000 rpm on a vitreous carbon electrode. Linear sweep excitations between 0.8 V and -0.5 V vs. a silver/silverchloride/potassiumchloride reference electrode and a scan rate of -40 mV s^{-1} are performed for all samples. During the reduction process, a diffusion layer of constant thickness is formed by the forced convection [10–12]. Constant reduction currents result for half-cell potentials < -0.1 V vs. Ag/AgCl/KCl(sat.), which are potential independent and purely represent mass transport limitation by the educt from the bulk phase [11,12]. According to Levich [10–13], reduction currents are directly proportional to the concentration c(Br_2) of the bulk solution.

Calibration of this methode and calculation of Br_2 concentrations of aqueous electrolytes are described in detail in ref. [4]. As Küttinger et al. [4] describe, picture and tabulate the bromine concentrations of the aqueous phases of the corresponding fused salts here, the tabulated values of Br_2(aq) concentrations are applied in this work from the ESI of ref. [4].

1.6. Concentration of $[BCA]^+$ cations in fused salt phases

The $[BCA]^+$ cations are transferred from the aqueous electrolyte phase into the fused salt phase followong the complexing equilibrium of Eqn 1 (research article). Concentrations of 1-alkyl-3-methylimdiazol-1-ium cations and 1-alkylpyridin-1-ium cations are calculated from volumes of the fused salt phase V(fs) and the aqueous electrolyte phase V(aq), the total $[BCA]^+$ concentration of the sample (1.11 M) and the $[BCA]^+$ concentrations of the aqueous electrolyte phase followiing Eqn. S3:

Eqn. S3 $$c([BCA]^+, fs) = \frac{1.11\ M \cdot 0.03\ L}{V(fs)} - \frac{c([BCA]^+,\ aq) \cdot V(aq)}{V(fs)}$$

Concentrations of 1-alkylpyridin-1-ium cations and 1-alkyl-3-methylimidazol-1-ium cations in aqueous electrolyte phase c($[BCA]^+$, aq) are determined by Raman spectroscopy. Both cations show characteristic strong peaks at distinctive raman shifts especially due to their aromaticity. Characteristic Raman shifts of peaks of the BCAs, the application of the peaks, the caluclation of the concentrations of c($[BCA]^+$, aq) from the peaks and values of c($[BCA]^+$, aq) are shown in ref. [4] in detail. The tabulated values of c($[BCA]^+$, aq) are applied in Eqn. S3 to calculate the $[BCA]^+$ concentrations in the fused salt.

1.7. Polybromide determination and Br_2 distribution on the polybromides in fused salt phases

1.7.1. Raman activity of the polybromides and determination of the stored Br2 fractions in the fused salt

Br_2 is bound with bromide in form of different polybromides like tribromide Br_3^-, pentabromide Br_5^- and heptabromide Br_7^- in aqueous phases and fused salt phases [16,59–61]. Symmetrical and antisymmetrical stretching vibrations in polybromides occur between the inner bromide ion and the outer atoms of the Br_2 molecule attached via an addition bonding [4,9,14] when applying Raman spectroscopy to polybromides in various solutions. For the three mentioned polybromides and pure Br_2 this results in Raman spectra with different Raman shifts of symmetrical and antisymmetrical streching vibrations, depending on the polybromide in the range of Raman shifts between $\tilde{\nu}$ = 150 and 320 cm^{-1} as described in literature [4,9,14–16]. Obtained Raman spectra of fused salt phases of the electrolytes in this study are corrected in the Rayleigh range and the peak areas of the following stretching oscillations of the polybromides are determined by fitting according to the Lorentz model and using the iteration algorithm according to Levenberg-Marquardt: $\tilde{\nu}(Br_3^-,\ sym.) \approx$ 164-170 cm^{-1} [14,15,17], $\tilde{\nu}(Br_3^-,\ antisym.) \approx$ 190-

198 cm^{-1} [14,15,18], ṽ(Br_5^-, antisym.) ≈ 210 cm^{-1} [14,15], ṽ(Br_5^-, sym.) ≈ 253-255 cm^{-1} [14,15,18–20], ṽ(Br_7^-, sym.) ≈ 269 cm^{-1} [14,19]. In the literature, pure Br_2 shows a strong single peak at ṽ(Br_2,sym.) ≈ 300-325 cm^{-1} [18–20]. Since there are differences in Raman shifts between aqueous solution and the corresponding fused salt phase, the Raman shifts of the symmetrical streching vibrations of Br_3^-, Br_5^- and Br_7^- are shown in Table 2 in the research article for the fused salt phase. Comparison with the Raman shifts of the same polybromides in the aqueous phase is possible taking ref. [4] into account. In the Raman spectra due to the addition of two Br_2 molecules to a bromide, twice the intensity per Br_5^- compared to a Br_3^- anion can be expected for a Br_5^- molecule. For Br_7^- the intensity is expected to be three times as high as for Br_3^- anions. The areas under the curves of the symmetric stretching oscillation correspond to the sum of the Br_2 molecules involved in this stretching vibration of one polybromide type. The fraction of the area of a symmetrical stretching oscillation in relation to the sum of all areas of the symmetrical stretching oscillations of Br_3^-, Br_5^- and Br_7^- gives the fraction of the Br_2 concentration present in each polybromide x(Br_3^-), x(Br_5^-) and x(Br_7^-). A detailed description and explanation of the method is printed in [9]. All Raman spectra of the aqueous phase are shown in Figure S1-S7 in this ESI.

1.7.2. Raman equipment and sample treatment

By means of Raman spectroscopy on the fused salt phase samples, the occurrence of the different polybromides tribromide Br_3^-, pentabromide Br_5^- and heptabromide Br_7^- in fused salt series of 1-alkylpyridin-1-ium cations ($[C2Py]^+$, $[C4Py]^+$, $[C6Py]^+$) und 1-alkyl-3-methylimidazol-1-ium cations ($[C2Mim]^+$, $[C3MIm]^+$, $[C4MIm]^+$ and $[C6MIm]^+$) at different SoCs (10, 20, 30, 33, 40, 50, 60, 66, 70, 80, 90, 100 %) are investigated. Raman spectra are measured with the HORIBA LabRAM HR spectrometer (JOBIN YVON Technology GmbH, Germany) at room temperature ϑ = 23 ± 1 C in the wavenumber (ṽ) range from ṽ = 50 cm^{-1} to 4000 cm^{-1}. A frequency-doubled Nd:YAG laser (neodymium-doped yttrium-aluminium-garnet laser) with an excitation wavelength of λ = 532 nm (green) is used as laser source and a charge coupled device camera detector (CCD) at ϑ = -70 °C. Equipment and procedure is similar to ref. [4,9]. Samples of the liquid fused salts are pipetted into a quartz glass cuvette (Suprasil QS, Helma), sealed and examined under the Raman microscope. The focal point is positioned just below the edge of the front glass of the cuvette and the position of the carrier table is fixed to obtain reproducible Raman spectra. For all samples and Raman shifts, the exposure time is 10 s and the number of repetitions per sample for calculating the average is set to three.

1.8. Electrolytic conductivities of fused salt catholyte

Ionic conductivities of the fused salt phase of 1-alkylpyridin-1-ium BCAs and 1-alkyl-3-methylimidazol-1-ium BCAs electrolyte mixtures are determind. The fused salt of different BCAs are investigated at various SoC (10, 20, 30, 33, 40, 50, 60, 66, 70, 80, 90 and 100 %) and at at ϑ = 23 ± 1°C. In the conductivity cell LF 1101 (SI Analytics GmbH/Germany) ohmic electrolyte resistances $R_{ELECTROLYTE(measurement)}$ are measured by help of potentiostatic impedance spectroscopy with a perturbation of û = 10 mV (amplitude) in the frequency range between 1 MHz and 100 Hz, without a potential offset. Ohmic resistances $R_{ELECTROLYTE(measurement)}$ from the measurement are corrected by the ohmic resistance of cables and connections to gain $R_{ELECTROLYTE(fs)}$. Cell constants $K_{Conductometer}$ are determind for each row of 12 measurements in a 1 M KCl solution at ϑ = 23 ± 1 °C. The conductivity of 1 M KCl solution at ϑ = 23 ± 1 °C is determined by linear regression from literature [21] to be κ(1 M KCl, ϑ = 23 °C) = 103.9 mS cm^{-1}. Between the measurements the sensor was rinsed first with acetone and after with purified water. $K_{Conductometer}$ and electrolyte conductivity $\kappa_{ELECTROLYTE(fs)}$ are calculated by means of Eqn. S4 and Eqn. S5:

Eqn. S4 $$\kappa_{\mathrm{ELECTROLYTE(fs),\ t=23°C}} = \frac{1}{\mathrm{R}_{\mathrm{Electrolyte,\ t=23°C}} \cdot \mathrm{K}_{\mathrm{Conductometer}}}$$ [κ] = S cm^{-1}

Eqn. S5 $$K_{Conductometer} = \frac{A}{l} = \frac{1}{R_{1M\,KCl,\;t=23°C} \cdot \kappa_{1M\,KCl,\;\vartheta=23°C}}\ [K] = cm$$

1.9. Rheological behaviour of fused salt phase

Rheological data of liquid fused salts of 1-alkylpyridin-1-ium BCAs and 1-alkyl-3-methylimidazol-1-ium BCAs are measured at various SoC using MCR302/Anton Paar rheometer, with plate/plate configuration (PP50/CX). Both plates of the plate/plate system are PTFE coated. Shear rates of 10 up to 100 s^{-1} at ϑ = 23 ± 1 °C with a distance of 0.5 mm between the plates are applied in the measurements. Results (shear stress vs. shear velocity) are revised on linear viscous flow behavior and dynamic shear viscosities η are calculated.

1.10. Temperature stability of the two phase electrolyte

All investigations in this work have been carried out at room temperature ϑ = 23 ± 1 °C. Both, aqueous electrolyte phase and fused salt can crystallise at lower temperatures. Using the example of the electrolyte with [C2Py]Br as BCA, the electrolyte samples of different SoCs (0, 10, 20, 30, 33, 40, 50, 60, 66, 70, 80, 90 and 100 %) are cooled down in a climatic chamber from ϑ = 25 °C in steps of $\Delta\vartheta$ = -1 °C up to a maximum of ϑ = -22 °C and visually examined whether and which of the two electrolyte phases crystallizes. Samples are kept at the respective temperature for at least 3 hours before investigation.

2. ^{1}H NMR and ^{13}C NMR characterization of synthesized bromine complexation agents.

The BCAs synthesized have already been prepared in our work for investigating the aqueous phase with the same BCAs. The NMR spectra are therefore taken from reference [4], but characterize the structure of the used BCAs used in this article:

^{1}H NMR and ^{13}C NMR spectra are recorded as shown below and confirmed by existing literature the synthesis of the expected BCAs [3,5–8,22,23]. ^{1}H NMR and ^{13}C NMR spectra are measured on a 400 MHz Bruker AV-400 spectrometer (400 MHz for ^{1}H NMR and 100 MHz for ^{13}C NMR) using DMSO-d_6 as solvent. The ^{1}H NMR data are reported as follows in ppm (δ) from the internal standard (TMS, 0.0 ppm); chemical shift (multiplicity, coupling constant in Hz, integration), and the ^{13}C NMR data in ppm (δ) from the internal standard (TMS, 0.0 ppm). NMR spectra are only examined for chemicals listed in Table 1 of the manuscript which are not purchased commercially. These chemicals are listed in the "Experimental Section" in the sub-item "Synthesis of bromine complexing agents" of the manuscript:

1-ethylpyridin-1-ium bromide [C2Py]Br

^{1}H NMR (400 MHz, DMSO-d_6) δ(ppm): 1.53 (t, J = 7.2 Hz, 3H), 4.69 (q; J = 7.3 Hz, 2H), 8.18 (t, J = 7.1 Hz, 2H), 8.62 (t, J = 7.8 Hz, 1H), 9.10-9.53 (m, 2H). ^{13}C NMR (100 MHz, DMSO-d_6) δ(ppm): 16.38, 56.19, 128.02, 144.55, 145.39.

1-n-butylpyridin-1-ium bromide [C4Py]Br

^{1}H NMR (400 MHz, DMSO-d_6) δ(ppm): 0.89 (t, J = 7.2 Hz, 3H), 1.16-1.33 (m, 2H), 1.74-1.94 (m, 2H), 4.58 (t; J = 7.4 Hz, 2H), 8.15 (t, J = 7.1 Hz, 2H), 8.63 (t, J = 7.8 Hz, 1H), 9.12-9.47 (m, 2H). ^{13}C NMR (100 MHz, DMSO-d_6) δ(ppm): 13.31, 18.69, 32.67, 60.33, 128,04, 144,76, 145.

1-n-hexylpyridinium bromide [C6Py]Br

^{1}H NMR (400 MHz, DMSO-d_6) δ(ppm): 0.83 (t, J = 7.5 Hz, 3H), 1.12-1.37 (m, 6H), 1.80-1.98 (m, 2H), 4.57 (t; J = 7.6 Hz, 2H), 8.20 (t, J = 7.0 Hz, 2H), 8.60 (t, J = 7.7 Hz, 1H), 9.05-9.40 (m, 2H). ^{13}C NMR (100 MHz, DMSO-d_6) δ(ppm): 13.77, 21.80, 24.97, 30.50, 30.68, 60.54, 128.04, 144.75, 145.47.

1-ethyl-3-methylimidazol-1-ium bromide [C2MIm]Br

^{1}H NMR (400 MHz, DMSO-d_6) δ(ppm): 1.40 (t, J = 7.3 Hz, 3H), 3.86 (s; 3H), 4.21 (q, J = 7.3 Hz, 2H), 7.81 (t, J = 1.9 Hz, 2H), 9.31 (s, 1H). ^{13}C NMR (100 MHz, DMSO-d_6) δ(ppm): 15.14, 35.70, 44.06, 121.93, 123.48, 136.24.

1-n-propyl-3-methylimidazol-1-ium bromide [C3MIm]Br

^{1}H NMR (400 MHz, DMSO-d_6) δ(ppm): 0.84 (t, J = 7.5Hz, 3H), 1.71-1.86 (m, 2H), 3.87 (s; 3H), 4.15 (t, J = 7.1 Hz, 2H), 7.79 (t, J = 1.6 Hz, 2H), 9.30 (s, 1H). ^{13}C NMR (100 MHz, DMSO-d_6) δ(ppm): 10.38, 22.83, 35.73, 50.16, 122.23, 123.54, 136.5.

1-n-butyl-3-methylimidazol-1-ium bromide [C4MIm]Br

^{1}H NMR (400 MHz, DMSO-d_6) δ(ppm): 0.87 (t, J = 7.4 Hz, 3H), 1.21-1.37 (m, 2H), 1.70-1.88 (m, 2H), 3.86 (s, 3H), 4.18 (t; J = 7.3 Hz, 2H), 7.65 (t, J = 1.8 Hz, 2H), 9.15 (s, 1H). ^{13}C NMR (100 MHz, DMSO-d_6) δ(ppm): 13.25, 18.72, 31.34, 35.74, 48.40, 122.23, 123.55, 136.50.

1-n-hexyl-3-methylimidazol-1-ium bromide [C6MIm]Br

^{1}H NMR (400 MHz, DMSO-d_6) δ(ppm): 0.78-0.93 (m, 3H), 1.18-1.39 (m, 6H), 1.71-1.85 (m, 2H), 3.86 (s, 3H), 4.23 (t, J = 7.21 Hz, 2H), 7.41 (t, J = 1.7 Hz, 2H) 9.26 (s, 1H). ^{13}C NMR (100 MHz, DMSO-d_6) δ(ppm): 13.80, 21.84, 25.09, 29.33, 30.51, 35.74, 48.68, 122.22, 123,53, 136,49.

3. Results of the manuscript in tabulated form

Table S3. Concentration of Br_2 in the fused salt phase depending on SoC (mixture of the electrolyte) and the chosen BCA in mol L^{-1}. Values are calculated from the total sample volume, the fused salt phase volume of the sample, the aqueous electrolyte volume of the sample. In addition, total concentrations of Br_2 and Br_2 concentrations of the aqueous phase are used from ref. [4]. Values in brackets may be subject to errors, as the volumes of the fused salt phase are smaller than 2 mL and measurement uncertainties of the volumes lead to errors in the calculation.

SoC (%)	[C2Py]Br (fs)	[C4Py]Br (fs)	[C6Py]Br (fs)	[C2MIm]Br (fs)	[C3MIm]Br (fs)	[C4MIm]Br (fs)	[C6MIm]Br (fs)
10	(11.47)	(17.42)	(9.69)	-	(15.73)	(17.65)	(6.50)
20	(10.74)	(7.55)	(5.68)	-	(9.07)	(7.63)	(7.97)
30	10.56	5.73	4.98	-	7.05	6.47	4.98
33	11.77	6.37	5.05	-	7.33	6.40	5.06
40	10.38	6.32	5.62	-	7.60	6.43	5.67
50	10.19	7.26	6.33	9.24	7.88	7.33	6.46
60	10.02	7.59	7.00	10.23	8.54	7.96	7.00
66	10.97	8.67	7.74	10.30	9.37	8.65	7.78
70	10.53	9.19	8.15	10.04	9.55	9.20	8.17
80	11.26	9.71	8.72	10.55	10.45	9.73	8.66
90	11.93	10.36	8.78	11.73	11.02	10.24	9.76
100	12.47	10.44	9.48	12.22	13.30	10.94	9.32

Table S4. Concentration of $[BCA]^+$ cations in the fused salt phase depending on SoC (mixture of the electrolyte) and the chosen BCA in mol L^{-1}. Values are calculated from the total sample volume, the fused salt phase volume of the sample, the aqueous electrolyte volume of the sample. In addition, total concentrations of [BCA]Br and $[BCA]^+$ cation concentrations of the aqueous phase are used from ref. [4]. Values in brackets may be subject to errors, as the volumes of the fused salt phase are smaller than 2 mL and measurement uncertainties of the volumes lead to errors in the calculation.

SoC (%)	[C2Py]Br (fs)	[C4Py]Br (fs)	[C6Py]Br (fs)	[C2MIm]Br (fs)	[C3MIm]Br (fs)	[C4MIm]Br (fs)	[C6MIm]Br (fs)
10	(14.23)	(17.19)	(7.73)		(22.01)	(23.13)	(5.62)
20	(10.06)	(8.18)	(7.05)		(9.83)	(8.76)	(8.40)
30	9.33	6.26	5.94		7.23	6.73	6.02
33	11.06	6.17	5.61		7.27	6.65	5.48
40	9.12	6.11	5.58		6.77	6.12	5.71
50	7.52	5.86	5.33	6.79	6.13	6.06	5.33
60	6.59	5.54	5.00	6.55	5.98	5.55	5.00
66	6.64	5.59	5.00	6.29	5.98	5.63	4.99
70	6.20	5.63	5.00	5.89	5.67	5.55	4.95
80	5.81	5.18	4.70	5.39	5.47	5.18	4.70
90	5.39	4.93	4.26	5.39	5.18	4.93	4.70
100	5.03	4.69	4.05	5.03	5.55	4.64	3.99

Table S5. Fused salt volume fraction in the two phase electrolyte vs. the total volume of the electrolyte sample (aqueous and fused salt phase) depending on the SoC in % at $\vartheta = 23 \pm 1$ °C. Values in brackets may be subject to errors, as the volumes of the fused salt phase are smaller than 2 mL and measurement uncertainties of the volumes lead to errors in the calculation.

SoC (%)	[C2Py]Br (fs)	[C4Py]Br (fs)	[C6Py]Br (fs)	[C2MIm]Br (fs)	[C3MIm]Br (fs)	[C4MIm]Br (fs)	[C6MIm]Br (fs)
10	(1.5)	(1.4)	(2.8)	-	(1.4)	(1.4)	(4.2)
20	(4.4)	(7.1)	(9.7)	-	(5.7)	(7.1)	(6.9)
30	7.5	14.5	16.7	-	11.4	12.9	16.7
33	7.5	14.3	18.1	-	12.1	14.3	18.1
40	10.4	16.9	19.4	-	14.5	17.1	19.4
50	13.2	18.3	20.8	14.7	17.1	18.3	20.8
60	16.2	19.7	22.2	15.7	18.6	20.0	22.2
66	16.4	19.7	22.2	17.6	18.6	19.7	22.2
70	17.9	19.7	22.2	18.8	19.6	20.0	22.2
80	19.1	21.4	23.6	20.6	20.3	21.4	23.6
90	20.6	22.5	26.0	20.6	21.4	22.5	23.6
100	22.1	23.7	27.4	22.1	20.0	23.9	27.7

Table S6. Density of the fused salt phase for the different used [BCA]Br substances depending on the SoC at $\vartheta = 23 \pm 1$ °C in g cm^{-3}.

SoC (%)	[C2Py]Br (fs)	[C4Py]Br (fs)	[C6Py]Br (fs)	[C2MIm]Br (fs)	[C3MIm]Br (fs)	[C4MIm]Br (fs)	[C6MIm]Br (fs)
10	1.92	1.75	1.65	-	1.84	1.72	1.65
20	1.95	1.78	1.63	-	1.81	1.74	1.63
30	1.96	1.80	1.63	-	1.85	1.73	1.64
33	1.98	1.80	1.64	-	1.85	1.75	1.63
40	2.00	1.85	1.69	-	1.85	1.78	1.66
50	2.02	1.87	1.77	1.94	1.90	1.83	1.75
60	2.07	1.98	1.83	1.99	1.97	1.89	1.81
66	2.09	1.96	1.83	2.00	1.98	1.94	1.80
70	2.14	1.97	1.85	2.30	2.01	1.94	1.83
80	2.15	2.03	1.91	2.13	2.06	2.00	1.89
90	2.22	2.07	1.97	2.18	2.09	2.06	1.94
100	2.28	2.12	2.00	2.26	2.14	2.09	1.99

Table S7. Br_2 distribution on different polybromides of the fused salt: fraction of Br_2 in tribromide Br_3^- depending on chosen BCA and for electrolyte mixtures at different SoCs.

SoC (%)	[C2Py]Br (fs)	[C4Py]Br (fs)	[C6Py]Br (fs)	[C2MIm]Br (fs)	[C3MIm]Br (fs)	[C4MIm]Br (fs)	[C6MIm]Br (fs)
10	0.364	0.671	0.847	-	0.586	0.692	0.872
20	0.477	0.618	0.839	-	0.565	0.649	0.861
30	0.422	0.491	0.728	-	0.492	0.583	0.750
33	0.415	0.526	0.695	-	0.468	0.538	0.726
40	0.362	0.385	0.493	-	0.420	0.430	0.589
50	0.298	0.310	0.271	0.321	0.328	0.313	0.352
60	0.225	0.176	0.206	0.329	0.246	0.215	0.220
66	0.183	0.187	0.193	0.286	0.218	0.182	0.212
70	0.159	0.178	0.171	0.250	0.196	0.171	0.178
80	0.140	0.140	0.126	0.200	0.152	0.124	0.140
90	0.109	0.112	0.106	0.164	0.111	0.096	0.102
100	0.079	0.087	0.097	0.134	0.091	0.068	0.076

Table S8. Br_2 distribution on different polybromides of the fused salt: fraction of Br_2 in pentabromide Br_5^- depending on chosen BCA and for electrolyte mixtures at different SoCs.

SoC (%)	[C2Py]Br (fs)	[C4Py]Br (fs)	[C6Py]Br (fs)	[C2MIm]Br (fs)	[C3MIm]Br (fs)	[C4MIm]Br (fs)	[C6MIm]Br (fs)
10	0.636	0.277	0.111	-	0.349	0.249	0.111
20	0.451	0.300	0.124	-	0.367	0.278	0.113
30	0.484	0.401	0.201	-	0.417	0.328	0.208
33	0.492	0.367	0.246	-	0.439	0.360	0.228
40	0.529	0.453	0.405	-	0.479	0.437	0.341
50	0.569	0.490	0.540	0.561	0.537	0.511	0.499
60	0.595	0.499	0.568	0.556	0.570	0.550	0.556
66	0.597	0.502	0.561	0.574	0.565	0.550	0.555
70	0.586	0.504	0.555	0.589	0.561	0.543	0.553
80	0.570	0.452	0.518	0.586	0.535	0.524	0.517
90	0.523	0.401	0.438	0.559	0.496	0.488	0.457
100	0.434	0.317	0.3756	0.509	0.423	0.446	0.398

Table S9. Br_2 distribution on different polybromides of the fused salt: fraction of Br_2 in heptabromide Br_7^- depending on chosen BCA and for electrolyte mixtures at different SoCs.

SoC (%)	[C2Py]Br (fs)	[C4Py]Br (fs)	[C6Py]Br (fs)	[C2MIm]Br (fs)	[C3MIm]Br (fs)	[C4MIm]Br (fs)	[C6MIm]Br (fs)
10	0.000	0.052	0.042	-	0.065	0.059	0.017
20	0.072	0.083	0.037	-	0.069	0.073	0.026
30	0.093	0.108	0.072	-	0.090	0.089	0.042
33	0.094	0.107	0.059	-	0.093	0.102	0.047
40	0.109	0.161	0.102	-	0.101	0.133	0.071
50	0.133	0.200	0.189	0.119	0.135	0.175	0.149
60	0.180	0.325	0.226	0.115	0.184	0.236	0.223
66	0.220	0.311	0.245	0.140	0.217	0.268	0.234
70	0.255	0.318	0.275	0.162	0.242	0.286	0.269
80	0.290	0.408	0.356	0.213	0.313	0.352	0.343
90	0.368	0.486	0.456	0.277	0.393	0.416	0.441
100	0.487	0.596	0.527	0.357	0.487	0.486	0.526

Table S10. Ionic conductivity of the fused salt phase depending on state of charge (electrolyte mixture) and chosen BCA at ϑ = 23 ± 1 °C in mS cm^{-1}.

SoC (%)	[C2Py]Br (fs)	[C4Py]Br (fs)	[C6Py]Br (fs)	[C2MIm]Br (fs)	[C3MIm]Br (fs)	[C4MIm]Br (fs)	[C6MIm]Br (fs)
10	42.60	15.00	6.55	-	20.17	13.29	5.89
20	43.53	16.26	6.32	-	21.58	14.07	5.73
30	48.05	20.81	7.25	-	24.49	15.58	6.58
33	48.83	19.67	7.69	-	24.89	16.85	6.73
40	53.35	26.37	10.55	-	28.53	20.36	8.93
50	60.37	31.76	17.39	-	34.93	25.42	13.08
60	67.66	43.76	22.54	47.71	42.36	32.71	18.92
66	72.27	42.38	23.52	52.34	45.28	36.30	20.338
70	74.81	44.08	24.73	58.29	46.93	36.88	21.40
80	79.66	49.33	29.18	65.84	52.36	42.33	25.76
90	83.34	53.77	34.14	73.35	57.46	46.61	29.15
100	86.81	57.79	35.51	78.31	60.21	50.39	32.95

Table S11. Dynamic viscosity of the fused salt phases depending on the chosen BCA and the SoC (electrolyte mixture) at ϑ = 23 ± 1 °C in mPas.

SoC (%)	[C2Py]Br (fs)	[C4Py]Br (fs)	[C6Py]Br (fs)	[C2MIm]Br (fs)	[C3MIm]Br (fs)	[C4MIm]Br (fs)	[C6MIm]Br (fs)
10	-	30.77	48.63	-	25.58	28.05	51.70
20	14.00	29.90	48.82	-	24.07	27.34	50.00
30	12.96	26.05	45.25	-	22.15	25.99	46.73
33	13.03	26.75	44.20	-	21.94	24.77	46.04
40	11.91	21.52	37.46	-	20.51	22.65	41.47
50	11.61	20.08	28.38	-	18.27	19.79	33.45
60	10.70	15.99	24.68	16.35	16.41	16.90	24.40
66	9.27	16.20	23.78	12.50	15.15	15.76	24.38
70	10.04	15.78	22.49	11.51	14.52	15.25	24.27
80	9.11	14.34	20.82	10.53	13.33	13.67	20.93
90	8.75	13.52	18.71	8.89	12.20	12.04	18.48
100	8.16	11.98	17.38	8.33	11.00	11.32	16.99

4. Raman spectra of fused salt phases for different BCAs within the entire SoC range

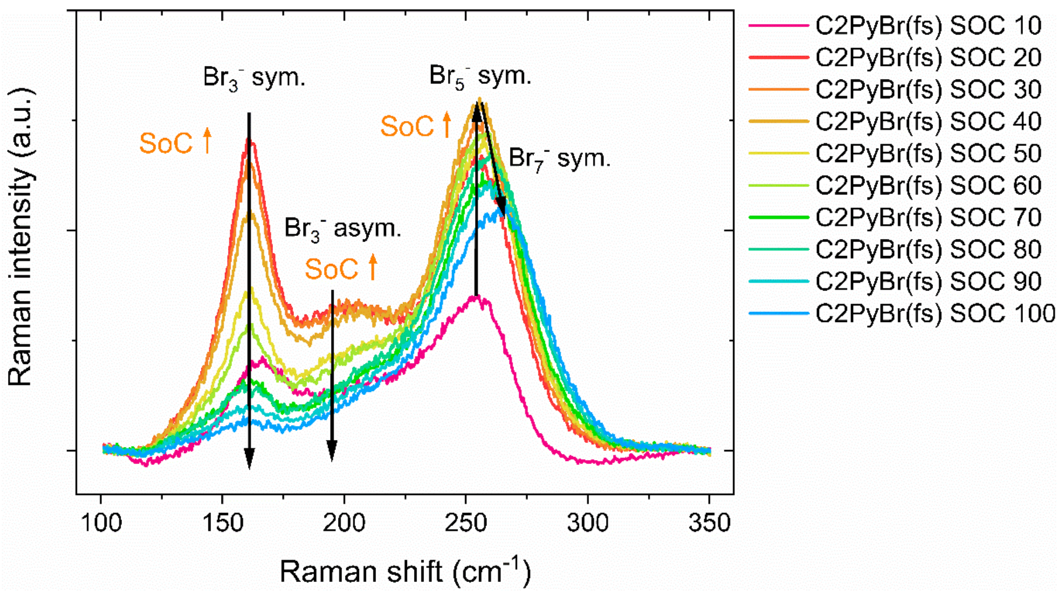

Figure S1. Raman spectra of fused salt phases with [C2Py]Br as BCA as a function of state of charge SoC.

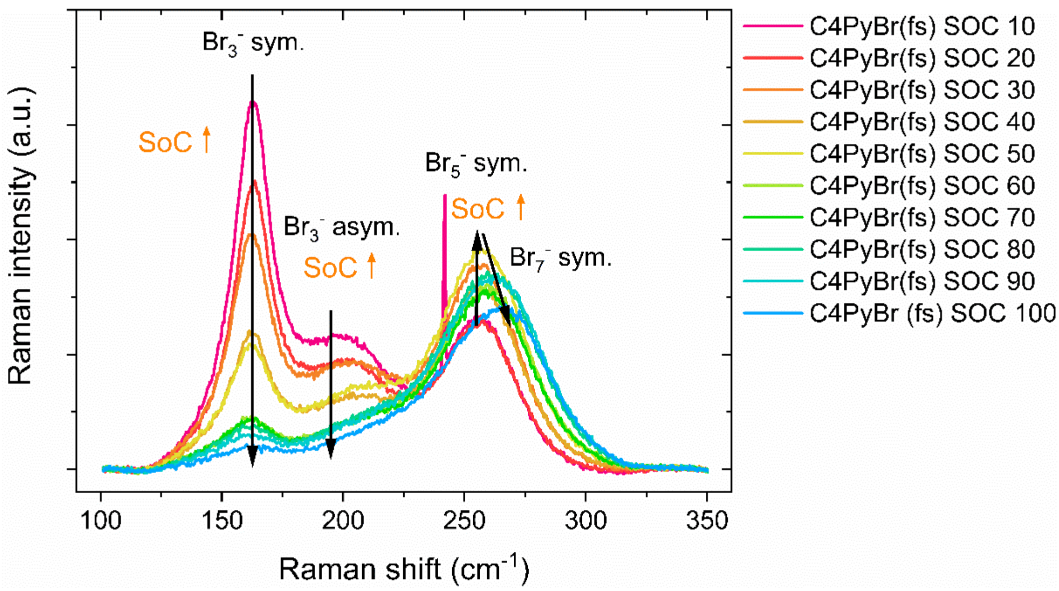

Figure S2. Raman spectra of fused salt phases with [C4Py]Br as BCA as a function of state of charge SoC.

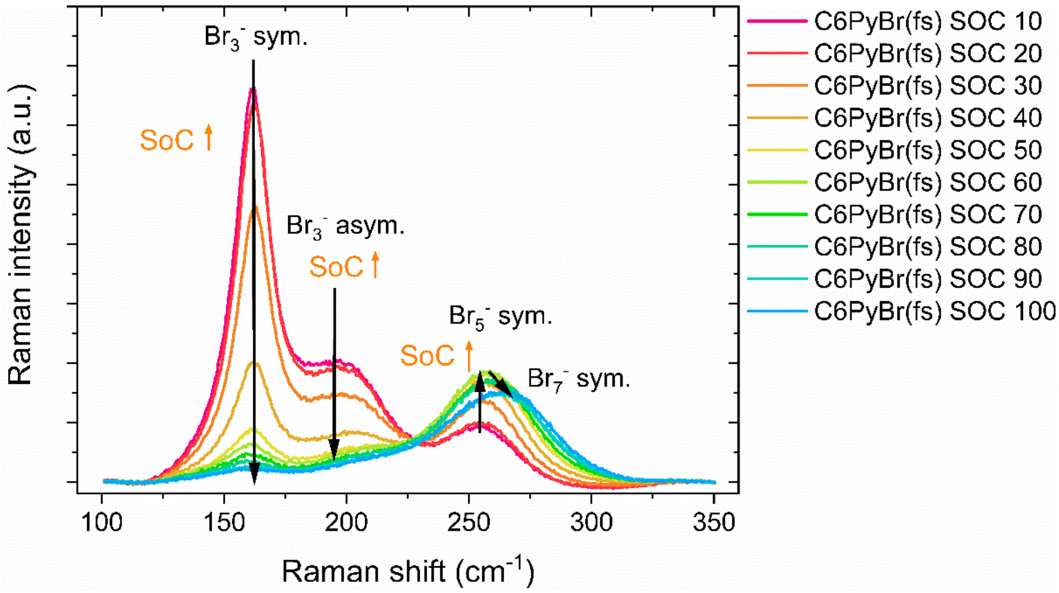

Figure S3. Raman spectra of fused salt phases with [C6Py]Br as BCA as a function of state of charge SoC.

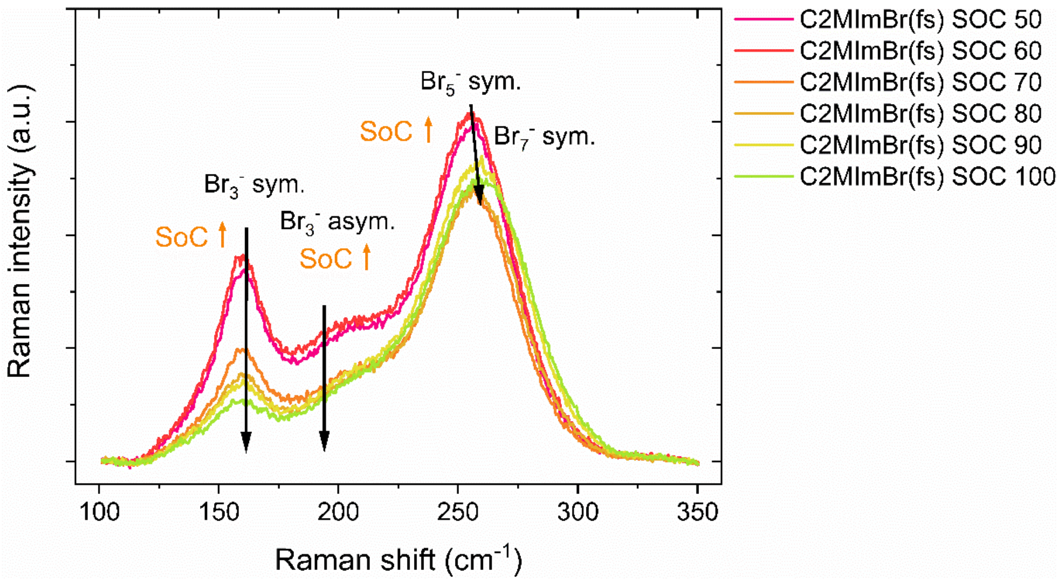

Figure S4. Raman spectra of fused salt phases with [C2MIm]Br as BCA as a function of state of charge SoC.

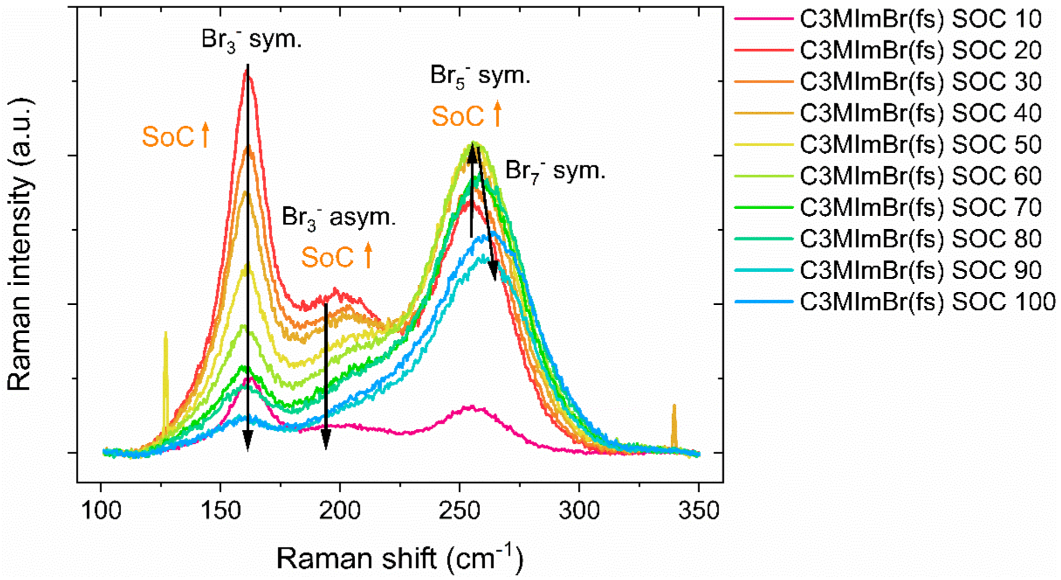

Figure S5. Raman spectra of fused salt phases with [C3MIm]Br as BCA as a function of state of charge SoC.

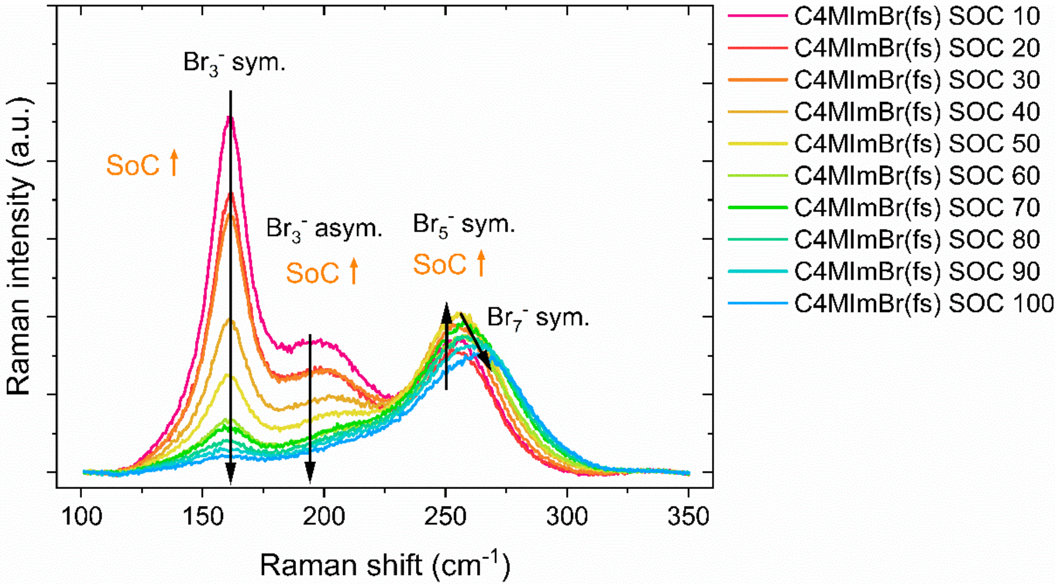

Figure S6. Raman spectra of fused salt phases with [C4MIm]Br as BCA as a function of state of charge SoC.

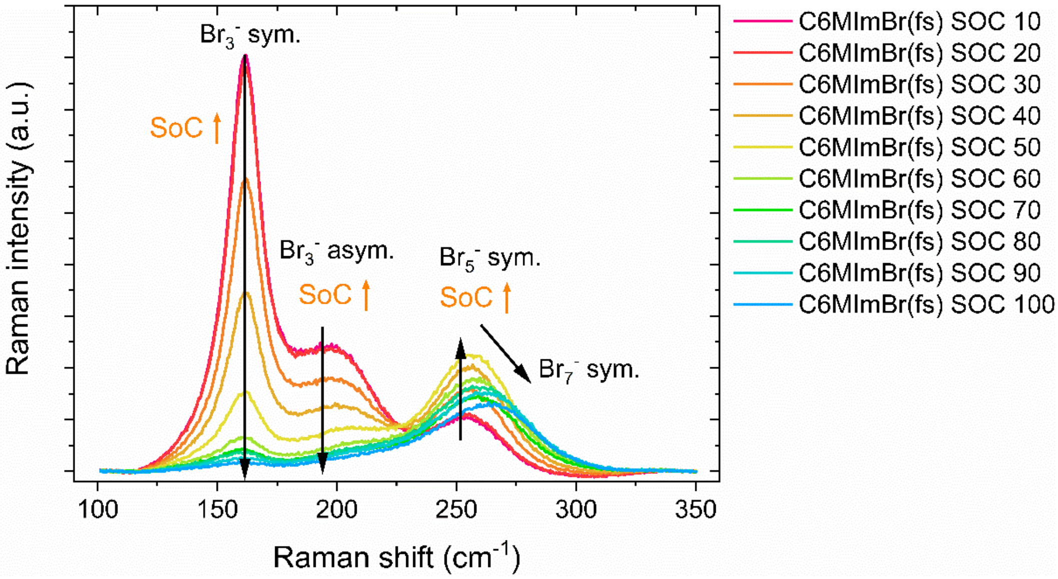

Figure S7. Raman spectra of fused salt phases with [C6MIm]Br as BCA as a function of state of charge SoC.

5 References of ESI

1. Dzyuba, S.V.; Bartsch, R.A. Efficient synthesis of 1-alkyl(aralkyl)-3-methyl(ethyl)imidazolium halides: Precursors for room-temperature ionic liquids. *J. Heterocycl. Chem.* **2001**, *38*, 265–268, doi:10.1002/jhet.5570380139.
2. Sashina, E.S.; Kashirskii, D.A.; Janowska, G.; Zaborski, M. Thermal properties of 1-alkyl-3-methylpyridinium halide-based ionic liquids. *Thermochim. Acta* **2013**, *568*, 185–188, doi:10.1016/j.tca.2013.06.022.
3. Aupoix, A.; Pégot, B.; Vo-Thanh, G. Synthesis of imidazolium and pyridinium-based ionic liquids and application of 1-alkyl-3-methylimidazolium salts as pre-catalysts for the benzoin condensation using solvent-free and microwave activation. *Tetrahedron* **2010**, *66*, 1352–1356, doi:10.1016/j.tet.2009.11.110.
4. Küttinger, M.; Loichet Torres, P.A.L.; Meyer, E.; Fischer, P.; Tübke, J. Systematic Study of Quaternary Ammonium Cations for Bromine Sequestering Application in High Energy Density Electrolytes for Hydrogen Bromine Redox Flow Batteries. *Molecules* **2021**, *26*, 2721, doi:10.3390/molecules26092721.
5. Bonhôte, P.; Dias, A.-P.; Papageorgiou, N.; Kalyanasundaram, K.; Grätzel, M. Hydrophobic, Highly Conductive Ambient-Temperature Molten Salts †. *Inorg. Chem.* **1996**, *35*, 1168–1178, doi:10.1021/ic951325x.
6. Alexey Deyko; Stephen G Hessey; Peter Licence; Elena A Chernikova; Robert G Jones. The enthalpies of vaporisation of ionic liquids: New measurements and predictions. *PCCP* **2012**, *14*, 3181–3193, doi:10.1039/c2cp23705a.
7. Butkene, R.V.; Mikul'skene, G.V.; ikher-Lorka, O.S.; Kupyatis, G.-K.K. Alkylation of 4-picolinium salts under phase transfer conditions. *Chem Heterocycl Compd* **1989**, *25*, 433–438, doi:10.1007/bf00480760.
8. Ghosh, R.; Ekka, D.; Rajbanshi, B.; Yasmin, A.; Roy, M.N. Synthesis,characterization of 1-butyl-4-methylpyridinium lauryl sulfate and its inclusion phenomenon with β-cyclodextrin for enhanced applications. *Colloids Surf., A* **2018**, *548*, 206–217, doi:10.1016/j.colsurfa.2018.01.003.
9. Küttinger, M.; Wlodarczyk, J.K.; Daubner, D.; Fischer, P.; Tübke, J. High energy density electrolytes for H 2 /Br 2 redox flow batteries, their polybromide composition and influence on battery cycling limits. *RSC Adv.* **2021**, *11*, 5218–5229, doi:10.1039/D0RA10721B.
10. Treimer, S.; Tang, A.; Johnson, D.C. A Consideration of the Application of Koutecký-Levich Plots in the Diagnoses of Charge-Transfer Mechanisms at Rotated Disk Electrodes. *Electroanalysis* **2002**, *14*, 165, doi:10.1002/1521-4109(200202)14:3<165:AID-ELAN165>3.0.CO;2-6.
11. Bard, A.J.; Faulkner, L.R. *Electrochemical Methods*: *Fundamentals and Applications*. Second Edition, 2nd edition; John Wiley & Sons, Inc.: United States of America, 2001, ISBN 978-0-471-04372.
12. Vielstich, W. Theorie und Anwendung der rotierenden Scheibenelektrode. *Z. Anal. Chem.* **1960**, *173*, 84–87, doi:10.1007/BF00448719.
13. Frumkin, A.; Nekrasov, L.; Levich, B.; Ivanov, J. Die Anwendung der rotierenden Scheibenelektrode mit einem Ringe zur Untersuchung von Zwischenprodukten elektrochemischer Reaktionen. *Journal of Electroanalytical Chemistry (1959)* **1959**, *1*, 84–90, doi:10.1016/0022-0728(59)80012-7.
14. Chen, X.; Rickard, M.A.; Hull, J.W.; Zheng, C.; Leugers, A.; Simoncic, P. Raman spectroscopic investigation of tetraethylammonium polybromides. *Inorg. Chem.* **2010**, *49*, 8684–8689, doi:10.1021/ic100869r.
15. Easton, M.E.; Ward, A.J.; Chan, B.; Radom, L.; Masters, A.F.; Maschmeyer, T. Factors influencing the formation of polybromide monoanions in solutions of ionic liquid bromide salts. *Phys. Chem. Chem. Phys.* **2016**, *18*, 7251–7260, doi:10.1039/c5cp06913k.
16. Easton, M.E.; Ward, A.J.; Hudson, T.; Turner, P.; Masters, A.F.; Maschmeyer, T. The formation of high-order polybromides in a room-temperature ionic liquid: from monoanions ([Br5](-) to [Br11

](-)) to the isolation of [PC16 H36]2 [Br24] as determined by van der Waals Bonding Radii. *Chem. Eur. J.* **2015**, *21*, 2961–2965, doi:10.1002/chem.201404505.

17. Hayward, G.C.; Hendra, P.J. The far infra-red and Raman spectra of the trihalide ions IBr−2 and I−3. *Spectrochimica Acta Part A: Molecular Spectroscopy* **1967**, *23*, 2309–2314, doi:10.1016/0584-8539(67)80124-7.
18. Evans, J.C.; Lo, Grace Y. S. Vibrational spectra of BrO-, BrO2-, Br3-, and Br5-. *Inorg. Chem.* **1967**, *6*, 1483–1486, doi:10.1021/ic50054a011.
19. Haller, H.; Schröder, J.; Riedel, S. Structural evidence for undecabromide [Br11]-. *Angew. Chem. Int. Ed.* **2013**, *52*, 4937–4940, doi:10.1002/anie.201209928.
20. Bauer, G.; Drobits, J.; Fabjan, C.; Mikosch, H.; Schuster, P. Raman spectroscopic study of the bromine storing complex phase in a zinc-flow battery. *J. Electroanal. Chem.* **1997**, *427*, 123–128, doi:10.1016/S0022-0728(96)04992-3.
21. *CRC handbook of chemistry and physics*: *A ready-reference book of chemical and physical data;* Haynes, W.M.; Lide, D.R., Eds., 96. ed., 2015-2016; CRC Press: Boca Raton, Fla., 2015, ISBN 978-1482260960.
22. Shimizu, Y.; Wachi, Y.; Fujii, K.; Imanari, M.; Nishikawa, K. NMR Study on Ion Dynamics and Phase Behavior of a Piperidinium-Based Room-Temperature Ionic Liquid: 1-Butyl-1-methylpiperidinium Bis(fluorosulfonyl)amide. *J. Phys. Chem. B* **2016**, *120*, 5710–5719, doi:10.1021/acs.jpcb.6b04095.
23. Yim, T.; Lee, H.Y.; Kim, H.-J.; Mun, J.; Kim, S.; Oh, S.M.; Kim, Y.G. Synthesis and Properties of Pyrrolidinium and Piperidinium Bis(trifluoromethanesulfonyl)imide Ionic Liquids with Allyl Substituents. *Bull. Korean Chem. Soc.* **2007**, *28*, 1567–1572, doi:10.5012/bkcs.2007.28.9.1567.
